# Midcell Recruitment of the DNA Uptake and Virulence Nuclease, EndA, for Pneumococcal Transformation

**DOI:** 10.1371/journal.ppat.1003596

**Published:** 2013-09-05

**Authors:** Matthieu J. Bergé, Alain Kamgoué, Bernard Martin, Patrice Polard, Nathalie Campo, Jean-Pierre Claverys

**Affiliations:** 1 Centre National de la Recherche Scientifique, LMGM-UMR5100, Toulouse, France; 2 Université de Toulouse, Université Paul Sabatier, Laboratoire de Microbiologie et Génétique Moléculaires, Toulouse, France; 3 Centre National de la Recherche Scientifique, LBME-UMR5099, Toulouse, France; University of Minnesota, United States of America

## Abstract

Genetic transformation, in which cells internalize exogenous DNA and integrate it into their chromosome, is widespread in the bacterial kingdom. It involves a specialized membrane-associated machinery for binding double-stranded (ds) DNA and uptake of single-stranded (ss) fragments. In the human pathogen *Streptococcus pneumoniae*, this machinery is specifically assembled at competence. The EndA nuclease, a constitutively expressed virulence factor, is recruited during competence to play the key role of converting dsDNA into ssDNA for uptake. Here we use fluorescence microscopy to show that EndA is uniformly distributed in the membrane of noncompetent cells and relocalizes at midcell during competence. This recruitment requires the dsDNA receptor ComEA. We also show that under ‘static’ binding conditions, i.e., in cells impaired for uptake, EndA and ComEA colocalize at midcell, together with fluorescent end-labelled dsDNA (Cy3-dsDNA). We conclude that midcell clustering of EndA reflects its recruitment to the DNA uptake machinery rather than its sequestration away from this machinery to protect transforming DNA from extensive degradation. In contrast, a fraction of ComEA molecules were located at cell poles post-competence, suggesting the pole as the site of degradation of the dsDNA receptor. In uptake-proficient cells, we used Cy3-dsDNA molecules enabling expression of a GFP fusion upon chromosomal integration to identify transformed cells as GFP producers 60–70 min after initial contact between DNA and competent cells. Recording of images since initial cell-DNA contact allowed us to look back to the uptake period for these transformed cells. Cy3-DNA foci were thus detected at the cell surface 10–11 min post-initial contact, all exclusively found at midcell, strongly suggesting that active uptake of transforming DNA takes place at this position in pneumococci. We discuss how midcell uptake could influence homology search, and the likelihood that midcell uptake is characteristic of cocci and/or the growth phase-dependency of competence.

## Author Summary

Natural genetic transformation, a programmed mechanism for horizontal gene transfer, permits the passage of environmental double-stranded (ds) DNA through the bacterial membrane and its subsequent integration into the recipient chromosome by homology. In the human pathogen *Streptococcus pneumoniae*, it requires development of a physiological state termed competence, which develops transiently in nearly all cells of an exponentially growing culture. Expression of a specific set of genes then allows assembly of a large membrane-associated machinery for binding exogenous dsDNA and internalizing single-stranded (ss) DNA fragments. The key role of converting dsDNA into ssDNA is fulfilled by EndA, a membrane-located endonuclease which is also a pneumococcal virulence factor pre-existing in noncompetent cells. Here, we report that EndA is uniformly distributed in the membrane of noncompetent cells and relocates into clusters during competence. We show that this relocalization is dependent upon the dsDNA-receptor ComEA and that ComEA and EndA are preferentially located at midcell in cultures exhibiting maximal transformation proficiency. Finally, using fluorescence microscopy, we visualize the transformation process in living cells providing evidence that DNA binding and presumably uptake occur at midcell.

## Introduction

Bacterial transformation, a programmed mechanism for genetic exchange, is based on the uptake and integration of exogenous DNA into the recipient genome. This exogenous DNA is captured in double-stranded (ds) form and internalized as single strands (ss). Gram-positive and Gram-negative microorganisms use related assemblies of proteins to internalize, protect and process transforming DNA [1]–[4]. Internalizing DNA is a complex process as it must cross the outer membrane (in Gram-negative bacteria), the cell wall and the cytoplasmic membrane. Uptake of exogenous DNA depends on the formation of a structure evolutionarily related to type IV pili and type-2 secretion systems [3], originally called the transformation pseudopilus [5]. A large macromolecular complex containing ComGC was found at the surface of competent *Bacillus subtilis* cells [6]. Most recently, a bona fide type IV transformation pilus was detected at the surface of competent *Streptococcus pneumoniae* [7]. Assembly of this pilus requires the traffic ATPase ComGA [5], [7]. Although the mode of action of the transformation pilus is still poorly understood, it binds DNA [7] and is known to be required for access of exogenous dsDNA to its membrane-bound receptor, ComEA, DNA binding being abolished in *comGA* mutant cells [8]. While evidence that DNA enters the cell in ss form has been obtained for only a very small number of species, all models for DNA uptake postulate entry of ssDNA into the cytosol through a transmembrane channel formed by ComEC [9] with ComFA acting as a translocase [10]. However, it is only with the Gram-positive human pathogen *S. pneumoniae* that the protein responsible for converting dsDNA into ssDNA is known. This sequence non-specific endonuclease, EndA, was the first component of the DNA-uptake apparatus identified [11]. This membrane protein [12], [13], is not required for initial dsDNA binding [11] but was proposed to degrade the non-transported strand [13]. Consistent with this role, degradation and uptake were shown to proceed with opposite polarities, and occur at similar rates (∼100 nt s^−1^ at 31°C), suggesting the functional coupling of degradation of one strand with import of its complement [14]. EndA is also a virulence factor; it enables pneumococci to escape the innate host immune response by degrading the DNA-scaffold trap elaborated by neutrophils [15]. EndA pre-exists in cells at the time they abruptly and simultaneously develop the ability to internalize ssDNA or competence.

Competence is induced in nearly all cells of an exponentially growing culture in response to a peptide pheromone, CSP (competence-stimulating peptide) [16], for a period of time as short as ∼20 min [17]. CSP induction ultimately activates the synthesis of the *comX* encoded competence-specific alternative σ^X^ factor [18] required for expression of the late competence (*com*) genes including those that encode the transformation machinery [19], [20]. The fact that EndA, uniquely among proteins of the uptake apparatus, is already present in cells prior to competence raises the question of its subcellular localization in noncompetent cells and its recruitment to the transformation machinery. Previous experiments had indicated that EndA-dependent degradation required the presence of the transformation pilus and the dsDNA receptor ComEA but not the transmembrane channel ComEC or the putative translocase ComFA [8]. Furthermore, *endA* mutant cells were found to accumulate DNA at the cell surface, presumably as a consequence of drastically reduced uptake. Here, we investigate EndA deployment and use it as a tool to document the subcellular localization of both bound transforming DNA and the DNA uptake machinery in competent pneumococci.

## Results

### EndA subcellular localization

**Figure 1:**
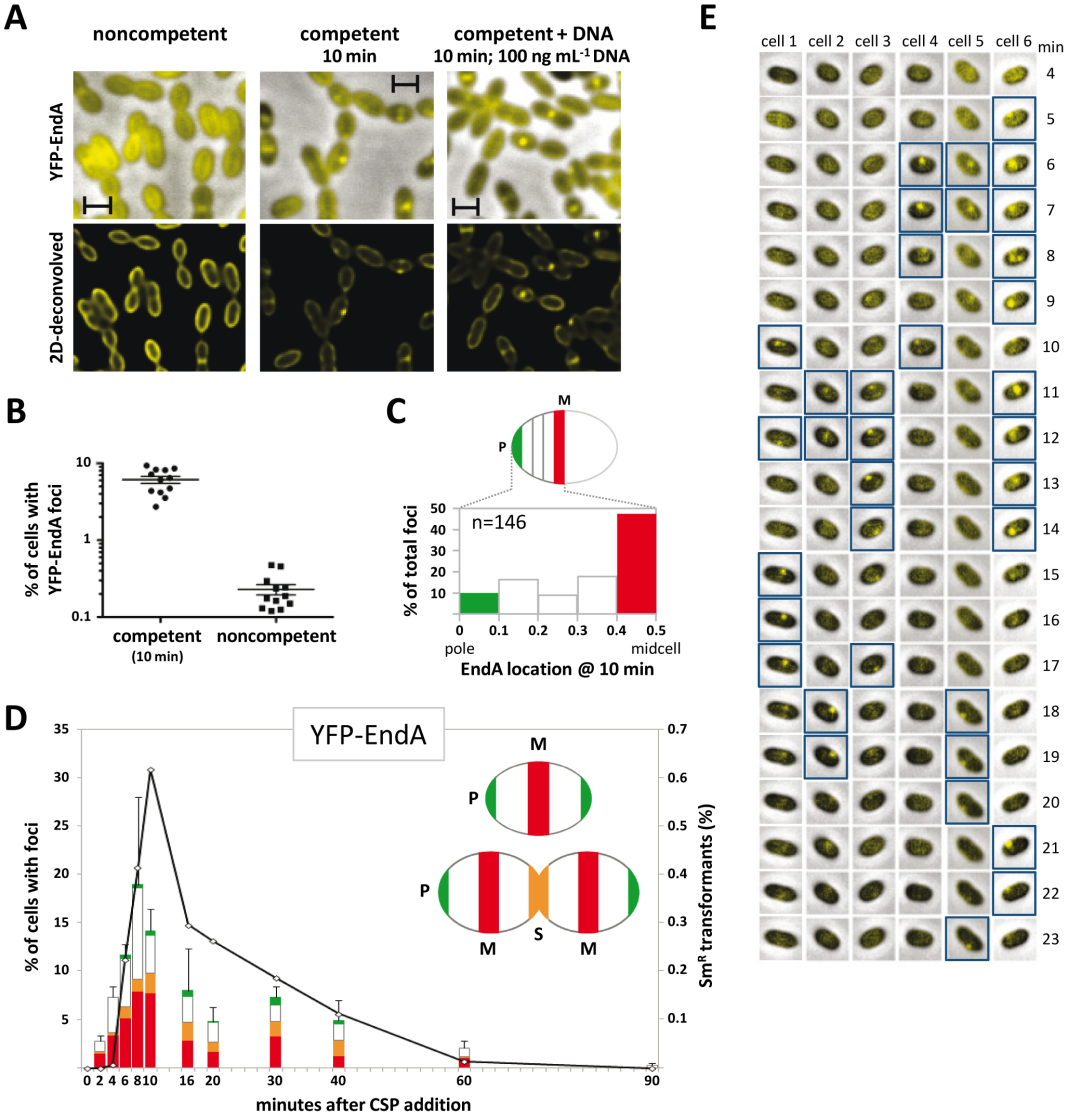
YFP-EndA clusters in competent pneumococci. (**A**) YFP-EndA distribution in cells 10 min after addition (or not - noncompetent) of CSP with or without R304 DNA. Top: overlay of phase-contrast images and YFP-EndA. Bottom: deconvolved fluorescent images (Materials and Methods) of YFP-EndA. Scale bars: 1 µM. (**B**) EndA clusters in a subset of the population. Data are mean ± s.d. of 4 fields of hundreds cells from 3 independent experiments (2,563 competent and 2,823 noncompetent cells analyzed). (**C**) Distribution of foci along longitudinal axis in cells without constriction. Focus (n = 146) position is given in relative coordinates from 0 (pole) to 0.5 (midcell). P, pole; M, midcell. (**D**) Transformation proficiency (open diamonds), fraction of cells containing foci and focus position (histograms). Transformation proficiency was assayed by selecting for Sm^R^ transformants as described in Materials and Methods. Color code (see inset cell diagram): red, midcell foci; white, intermediate position foci; orange, septal foci (S, septum); green, polar foci. (**E**) Fluorescence time-lapse microscopy of 6 competent YFP-EndA cells. Representative cells were extracted from a field containing 25 cells, all of which exhibited an EndA focus at some stage during the 25 minutes following CSP addition. Cells with foci are framed by blue squares.

We first compared the localization of EndA in competent and noncompetent cells using strains expressing EndA fluorescent protein fusions. The rationale for designing the fusions, the chromosomal location of chimeric genes, and the evaluation of functionality and impact on transformation frequency are summarized in Figure S1. Briefly, both GFP-EndA and YFP-EndA fusions were fully active, and modulation of their expression levels had no detectable effect on transformation frequency. Fluorescence microscopy analysis revealed that YFP-EndA is distributed all around the membrane in noncompetent cells (Figure 1A). Ten min after CSP addition and irrespective of the presence of exogenous DNA a different localization pattern emerged, with a number of cells exhibiting one or two discrete YFP-EndA foci (Figure 1A). Automated focus detection of over 2,500 cells using SpotFinder (MicrobeTracker image analysis software [21]) revealed that 6% of the cells contained foci, most of them (185/201) containing just one (Figure 1B). In cells without constriction (the most abundant cell type representing 77% of the population), about half the foci (47%) were at midcell (Figure 1C) and only 10% near a pole. Two widely different interpretations could account for the competence-dependent clustering of EndA. The straightforward explanation is that the protein is recruited at the entry pore as an active component of the uptake machinery. An alternative, which takes into account EndA's excess amount for transformation (Figure S1) and role as a virulence nuclease degrading dsDNA, would be that its clustering reflects the need to keep it away from the entry pore to protect transforming DNA.

### EndA clusters during DNA uptake window

Detection of foci in only a small fraction of the cells appeared paradoxical since all pneumococci in a culture are known to develop competence [22]. To determine whether this reflected brevity of focus life-time relative to the ∼20 min [17] competence period, we measured focus frequency as a function of time after CSP addition. This analysis revealed that the proportion of positive cells increased rapidly and reached a maximum (∼20%) 8 min after CSP addition, almost coinciding with maximal transformation proficiency in the population (Figure 1D). A 2-fold decrease was then observed over the next 10 min, and ∼60 min after CSP addition the frequency of cells with one focus was close to that measured in noncompetent cultures. The frequency of cells with foci paralleled the kinetics of transformation, suggesting a link between the capacity of YFP-EndA to concentrate into foci and DNA-uptake potential.

Time-lapse microscopy analysis then indicated that foci eventually formed in all cells during the competence window but lasted for only a few minutes, sometimes reforming at a different position in the same cell (Figure 1E and Movie S1). The disappearance of foci followed by their reformation at a different position strongly suggests that at the single-cell level competence is lasting longer than the lifespan of an individual focus. This dynamic behavior of EndA is consistent with a model in which the nuclease is transiently recruited at specific locations during competence development. It is of note that relocalization appeared to involve a large fraction of EndA molecules, despite evidence that only a small fraction (<10%) of them is required for DNA uptake ([23] and Figure S1).

### ComEA is required for EndA clustering

To investigate whether competence-dependent localization of EndA required expression of a late *com* gene, we examined the distribution of YFP-EndA in a *comX* mutant strain after CSP addition. The subcellular localization of YFP-EndA appeared indistinguishable from that seen in noncompetent cultures with no sign of clustering (Figure S2A). To identify the late *com* gene involved, we investigated YFP-EndA localization in a series of mutants lacking components of the transformation machinery. YFP-EndA was still able to concentrate into foci 10 min after CSP addition in ∼5% of cells lacking ComGA, ComEC or ComFA, with foci preferentially localized at midcell (Figure S2, A and B), as observed in wildtype cells. In contrast, YFP-EndA remained uniformly distributed around the membrane and failed to form foci in the absence of ComEA (Figure S2A). To rule out the possibility that the failure to form foci was due to degradation of the fusion protein, we monitored YFP-EndA levels in all mutants. Immunoblot analysis demonstrated that the amount of YFP-EndA in the absence of ComX, ComGA, ComEC, ComFA or ComEA was similar to that in wildtype cells (Figure S2C). These results demonstrate that competence-induced clustering of EndA relies on the dsDNA receptor ComEA, and is independent of the presence of other components of the DNA uptake machinery such as the transformation pilus, the transmembrane channel or the DNA translocase.

### DNA binding occurs at midcell

**Figure 2:**
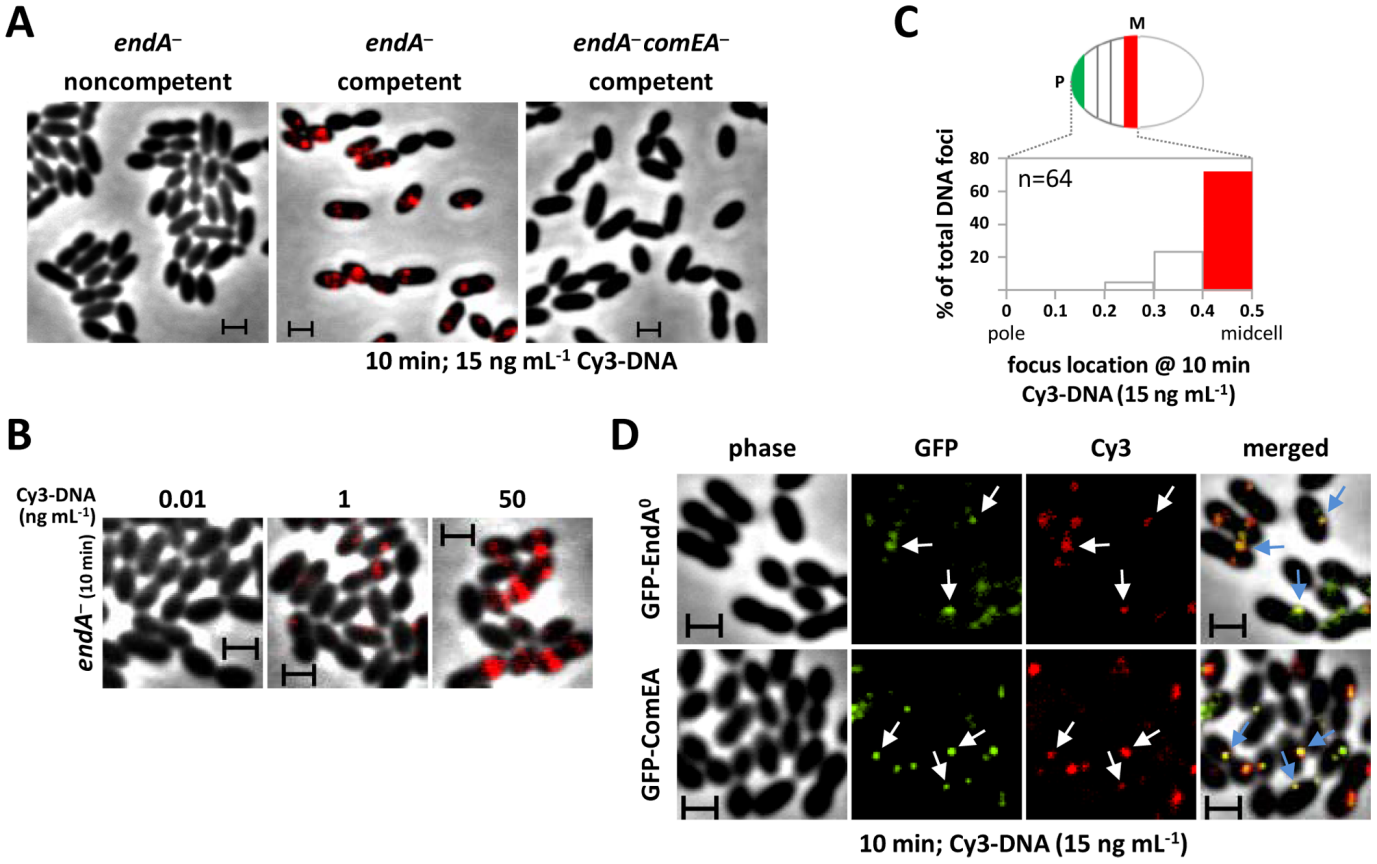
Bound transforming DNA, ComEA and EndA subcellular localizations. (**A**) Donor DNA binding at midcell in *endA* ^−^ cells. Fluorescent images of cells incubated with 285-bp Cy3-DNA. Strains: R2811, *endA^−^*; R3740, *endA^−^ comEA^−^*. (**B**) Effect of Cy3-DNA concentration on binding to *endA* ^−^ cells. (**C**) Distribution of Cy3-DNA bound to *endA^−^* cells. n, total number of DNA dots analyzed. See legend of Figure 1C for details. (**D**) Co-localization of Cy3-DNA with EndA or ComEA. Strains: R3741, *gfp-endA* ^0^; R3606, *gfp-comEA endA* ^−^ (GFP-ComEA fusion functionality assayed in Figure S3A). Arrows pinpoint the most prominent co-localization events. Scale bars: 1 µM.

To help distinguish between a clustering of EndA indicative of its recruitment to the DNA uptake machinery or sequestration away from the site of uptake, we investigated the location of DNA bound at the surface of competent pneumococci. We took advantage of the documented accumulation of DNA molecules at the surface of *endA^−^* cells, resulting from reduced uptake in the mutant [8]. Short DNA fragments (285 bp; to lessen spreading at the cell surface) harboring a single Cy3 label at the 5′ end (Cy3-DNA) were readily detected as fluorescent signals bound to competent *endA* ^−^ cells (Figure 2A). In contrast, we detected no fluorescence with noncompetent cells. Signal intensity was proportional to donor DNA concentration (Figure 2B), suggesting that the fluorescence detected represents bona fide competence-specific DNA binding. Further support for this conclusion was provided by the failure to detect bound Cy3-DNA in the absence of transformation pilus (i.e., in *endA comGA* mutant cells). In addition, no Cy3-DNA signal was observed with *endA comEA* double mutant cells (Figure 2A), strongly suggesting that DNA is retained at the surface of *endA* ^−^ cells through binding to the dsDNA receptor ComEA.

Bound Cy3-DNA was preferentially located at midcell (Figure 2C), with essentially no polar location detected, suggesting that the site of DNA binding could coincide with EndA location as determined in wildtype cells. To investigate the possible co-localization of EndA and bound DNA, we constructed a GFP fusion with an EndA protein devoid of nuclease activity, GFP-EndA^H160A^ (referred to as EndA^0^), based on the recent identification of the active site of EndA [24], [25]. Co-localization of Cy3-DNA and EndA^0^ was detected (Figure 2D). We also analyzed the possible co-localization of ComEA with Cy3-DNA using a strain harboring a functional GFP-ComEA fusion at the *comEA* locus. Similarly to EndA, ComEA frequently colocalized with DNA (Figure 2D). We conclude from these observations that midcell clustering of EndA corresponds to its recruitment to the DNA uptake machinery.

### ComEA life cycle

**Figure 3:**
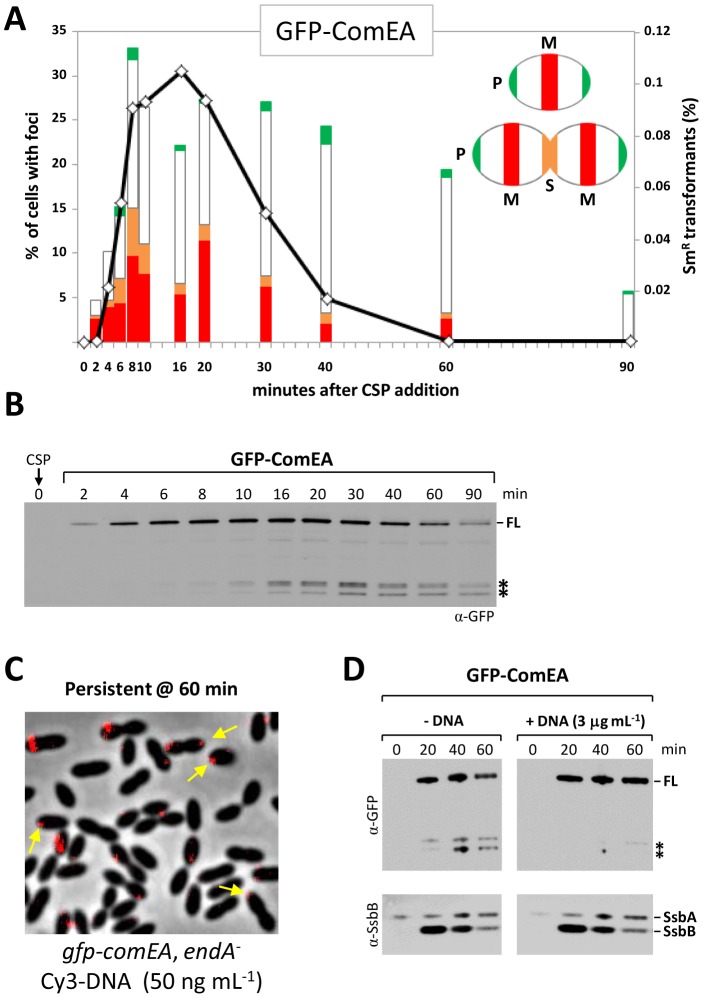
Localization and stability of GFP-ComEA. (**A**) Transformation proficiency (open diamonds), fraction of cells containing GFP-ComEA foci and focus position (histograms). See Figure 1D legend for details. (**B**) GFP-ComEA stability. Western-blot analysis of R2940 (*gfp-comEA*) extracts used anti-GFP antibodies. FL, full length fusion protein; asterisks, degradation products. (**C**) Persistence of Cy3-DNA fluorescence signal post-competence. Images of R3606 (*gfp-comEA endA* ^−^) cells 60 min after addition of CSP and 285 bp Cy3-DNA. Yellow arrows point to polar DNA molecules. (**D**) Stabilizing effect of persistent DNA on GFP-ComEA protein. Western-blot analysis of R3606 (*gfp-comEA*, *endA^−^*) cells incubated with or without R304 chromosomal DNA. Samples were collected at the indicated time points (see legend of panel B for details).

We then examined the subcellular localization of the GFP-ComEA fusion protein throughout a competence cycle (Figure 3A). As observed with EndA, ComEA appeared clustered in a subset of the competent population and the number of cells containing GFP-ComEA foci increased progressively until transformability reached a maximum in the culture. A closer examination of the positioning of EndA and ComEA foci suggested that these two proteins occupy similar locations at early time points following CSP addition (compare Figures 1D and 3A). Specifically, about half the first foci detected at 2 min post-CSP addition were close to midcell, consistent with emergence of foci at this position. This observation, together with the dependency of EndA clustering on ComEA and the co-localization of bound DNA with both proteins (Figure 2D), suggests a direct interaction of EndA and ComEA, an hypothesis which received indirect support (see Text S1).

However, ComEA and EndA did not colocalize permanently. Thus, while the fraction of cells containing EndA foci rapidly decreased 10 to 20 min after CSP addition and virtually disappeared from the culture by 60 min (Figure 1D), the fraction of cells containing GFP-ComEA foci remained steady until 40 min after CSP addition, and persisted in cells that were no longer competent (60 min time point; Figure 3A). A re-treatment of data shown in Figure 3A, involving recording also as polar foci those located in the section immediately next to the pole, revealed that the proportion of cells with GFP-ComEA foci near the poles increased significantly post-competence, i.e., 40 and 60 min after CSP addition (Figure S4). One interpretation of this localization pattern is that ComEA diffuses in the membrane from midcell toward the poles. Yet, ComEA did not accumulate further at the poles. Thus, the fraction of cells containing ComEA foci fell to 6% at 90 min (none with a focus at midcell) and vanished by 120 min, suggesting that ComEA could be progressively degraded once it reached the pole. Consistent with this hypothesis, increasing amounts of degradation products of GFP-ComEA protein appeared as competence declined (Figure 3B).

Post-competence ComEA, i.e., molecules present 60 min after CSP addition were unable to bind freshly added Cy3-DNA, possibly because of the disappearance of the transformation pilus. Nevertheless, a significant fraction of Cy3-DNA added early to *endA* ^−^ cells (i.e., at the time of CSP addition) remained bound to the surface after 60 min (Figure 3C). Examination of the location of this persistent material revealed again clear signs of migration toward the pole (Figure 3C, yellow arrows). The persistence of DNA bound to the cell surface was accompanied by stabilization of ComEA (Figure 3D), presumably a result of the interaction of the receptor with its dsDNA substrate.

### Rear-view detection of uptake

**Figure 4:**
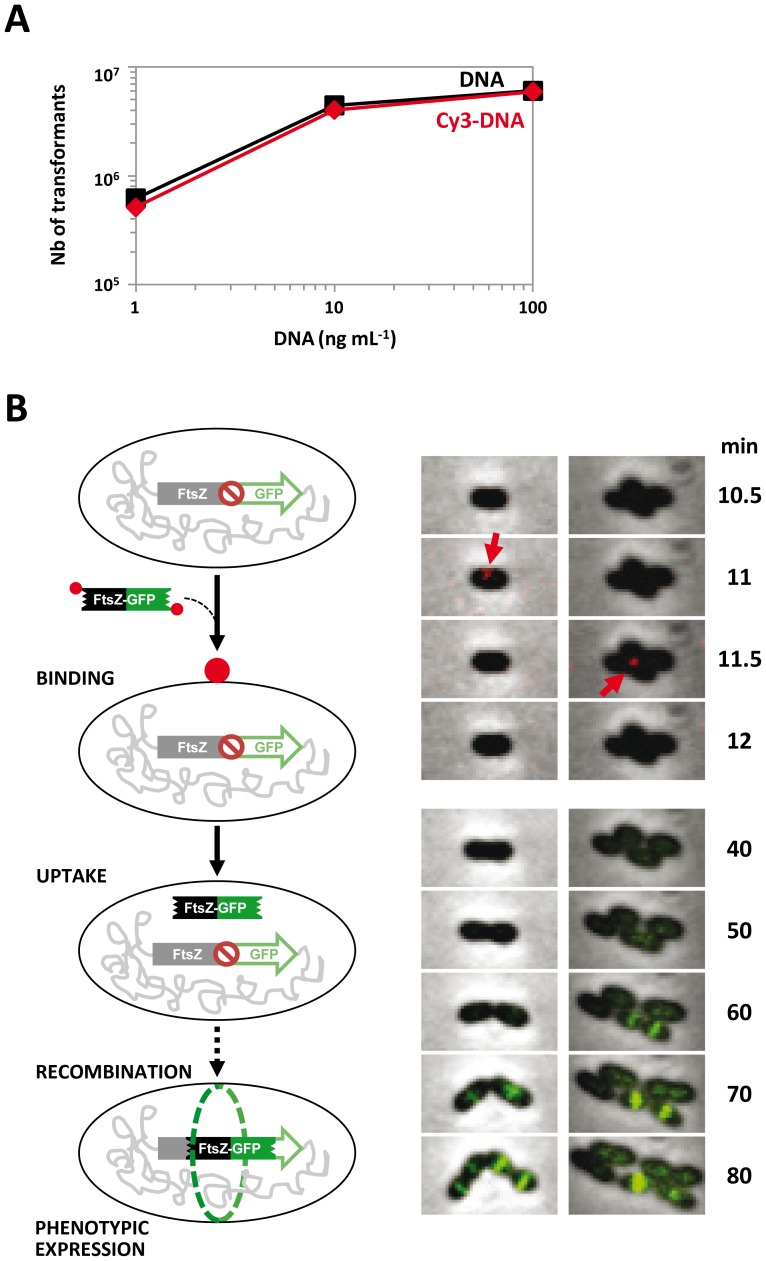
Direct visualization of transformation by fluorescence microscopy. (**A**) Transforming activity of Cy3-DNA. Wildtype cells were exposed to the indicated concentrations of a 4.3 kb fragment containing the Sm^R^ mutation *rpsL41*, bearing or not a 5′ Cy3 label, and Sm^R^ transformants were scored. Black, unlabelled donor DNA; red, Cy3-DNA. (**B**) Direct visualization of transformation. (*Left*) Schematic of transformation. Cy3-DNA, red dot; fluorescent FtsZ-GFP ring depicted in green. (*Right*) Fluorescence time-lapse microscopy. The Cy3 and GFP fluorescence images were respectively false colored red and green and overlaid on phase contrast images. Two representative cells are shown. Time after DNA addition indicated in min.

Evidence that DNA binding occurs at midcell was obtained under somewhat artificial conditions involving the use of *endA^−^* cells, which accumulate DNA molecules at the surface because of reduced uptake. We therefore wished to obtain evidence for midcell DNA binding in wildtype cells. Because multiple fluorophores internal to DNA are likely to interfere with its uptake [26], [27], and since uptake proceeds with 3′-5′ polarity [28], we first verified that pneumococcal cells could internalize DNA bearing a single Cy3 at its 5′ end. A Cy3-labelled 4.3-kb PCR fragment containing an Sm^R^ mutation transformed as efficiently as the unlabeled control (Figure 4A), indicating that the bulky terminal fluorophore did not interfere with uptake; this result did not, however, show that the fragment had been internalized with Cy3 still attached.

We then directly visualized the transformation process by fluorescence microscopy in living wildtype cells. The recipient strain harbored the *gfp* orf joined in frame to the 3′ end of *ftsZ* but separated from it by a stop codon. The Cy3-labelled donor fragment did not contain the stop codon, enabling expression of the FtsZ-GFP fusion upon integration into the chromosome (Figure 4B). Cy3-DNA was added to cells together with CSP and samples were taken 5 min later for recording at time intervals both DNA fluorescence and the eventual appearance of FtsZ-GFP. The latter allowed detection of transformed cells 60–70 min after sampling (Figures 4B and S5A). Looking back to the early period of DNA contact with these cells, we saw some Cy3-DNA foci at the surface 10–11 min post-sampling (Figures 4B and S5A). All foci were found exclusively at midcell, strongly suggesting that binding and presumably active uptake of transforming DNA takes place at this position in wildtype pneumococci. This interpretation was further supported by time-lapse microscopy of wildtype cells using Cy3-DNA fragments of widely different sizes (285 bp and 12 kb). Importantly, midcell fluorescence disappeared faster with the shorter fragment, which was detected at a single time-point, whereas the 12 kb fragment remained visible for about 2 min, a figure fully consistent with the rate of DNA uptake measured previously (∼100 nt s^−1^ at 31°C) [14] (Figure S5B). We conclude that the rapid disappearance of midcell fluorescence in wildtype cells results from active uptake of transforming DNA.

## Discussion

The EndA nuclease has a dual role in *S. pneumoniae*. It is a key component of the transforming DNA uptake machinery in competent cells [11] and a virulence nuclease [15]. It is unclear however how this membrane-bound protein [12], [13] fulfill the latter role since previous experiments showed a complete lack of degradation of transforming DNA added to comGA mutant cells [8]. This implied that despite its membrane location, EndA is unable to access exogenous DNA by itself. An explanation would be that EndA molecules responsible for degradation of the DNA-scaffold trap elaborated by neutrophils are not membrane-bound. Consistent with this view, cell-free filtrates of noncompetent *endA* ^+^ cultures were able to degrade DNA, strongly suggesting that some EndA molecules are liberated into the medium during normal growth [29]. EndA release could occur by autolysis, possibly subsequent to fratricide [30], [31], or by an unknown mechanism. The present study establishes two different subcellular localizations for EndA which is uniformly distributed in the membrane of noncompetent cells and relocalizes at midcell during competence. Recruitment at midcell is dependent on the dsDNA receptor ComEA. However, the observation that EndA no longer clusters in post-competent cells despite the persistence of ComEA foci suggests that ComEA alone might not be sufficient for clustering of the nuclease. Thus EndA clustering may require, in addition to ComEA, either competence-specific physiological conditions or another *com* gene product, different from the proteins constituting the transformation pilus, the transmembrane channel or the DNA translocase. Nevertheless, ComEA also localizes at midcell, together with bound dsDNA, and evidence suggests that transforming DNA uptake occurs at this position. It was suggested that EndA is fixed asymmetrically near the DNA entry pore and that degradation of the nontransported strand occurs by successive endonucleolytic cleavages [13]. While EndA cleaves ssDNA slightly faster than dsDNA in vitro [24], [25], the interaction with ComEA could modulate EndA activity so as to degrade only one strand to allow import of its complement. Alternatively, another component of the DNA uptake machinery could be responsible for the necessary adaptation of EndA activity to the uptake process.

Midcell uptake in *S. pneumoniae* contrasts with the situation in *B. subtilis* where several lines of evidence indicate that the subcellular site for active uptake is the cell pole [26], [32]–[35]. Proteins involved in integration of entering ssDNA into the *B. subtilis* chromosome, including the recombinase RecA, are also located at the pole. Extension towards the nucleoid of RecA nucleocomplexes formed at the pole is expected to be seen in this species, and threads emerging from polar RecA foci and extending into the cytosol were interpreted as such [32]. What could be the consequence of midcell uptake in *S. pneumoniae*? If accompanied by co-localization of RecA, this would result in formation of RecA nucleofilaments very close to the nucleoid. This situation may favor immediate inclusion of ssDNA-RecA complexes in the nucleoid, a ‘caging’ representing the most favourable situation for RecA-driven homology search. This suggestion is based on previous findings that an increase in DNA concentration (including in the form of completely heterologous DNA) accelerates homologous pairing through facilitated diffusion within nucleoprotein networks, also known as intersegmental homology search [36], [37]. Midcell uptake in *S. pneumoniae* could thus help accelerate chromosomal integration of internalized ssDNA, contributing to the rapid kinetics of a process known to be entirely completed within ∼10 min [1], in a species in which competence develops suddenly and disappears almost as rapidly owing to intrinsic shut-off mechanisms [38], [39].

What could account for the different locations of the uptake machinery in *B. subtilis* and *S. pneumoniae*? One major difference is seen in the growth-phase dependence of spontaneous competence development. The two species have evolved specific regulatory cascades, best adapted to their lifestyles, for tightly controlling assembly of orthologous DNA-uptake machines [40]. Under laboratory conditions, these cascades lead to competence induction during the transition to stationary phase in *B. subtilis* cultures, whereas pneumococcal competence develops in early exponential growth phase, i.e., in actively dividing cells. These differences could introduce species-specific constraints that account for the different implantation of transformation machineries. Another noticeable difference between these two bacteria is their shape. *B. subtilis* is a straight-rod shaped bacterium whereas *S. pneumoniae* is a rugby-ball shaped coccus. The morphology of bacterial cells is ultimately determined by the peptidoglycan cell wall. Large transenvelope complexes such as the transformation machinery presumably require the formation of gaps in the peptidoglycan for their assembly. An attractive idea is that remodeling by lytic enzymes [41], [42] occurring at the site of nascent peptidoglycan synthesis enables assembly of this apparatus. In support of this hypothesis, peptidoglycan synthesis occurs at midcell in actively growing pneumococci [43]. In *B. subtilis*, since competence develops at the onset of stationary phase, when the rate of peptidoglycan synthesis is slowing [44], it is tempting to speculate that the transformation machinery assembles at the septum during the last division cycle. Subsequent daughter cell separation would then result in the observed location, thus predicted to correspond to new poles. This interpretation would fit nicely with a recent report that ComN, which is involved in posttranscriptional control of *comEA* and *comEC* expression [45], localizes in a DivIVA-dependent manner at midcell where it promotes accumulation of the corresponding mRNA [46]. While the biological significance of this observation could not be established, the intriguing possibility that ComN could be required for proper localization of ComEC was not ruled out [46]. Nevertheless, the observation of competence complexes at both poles in *B. subtilis* cells and of polar complexes at non-adjacent poles in divided cells that have not separated (doublets) argues against assembly of the machinery at the septum during the last division. On the other hand, the existence of a significant fraction of non-polar foci (up to 30%) was reported in *B. subtilis* [26], [35] and it remains unclear whether they represent intermediates moving toward the poles for subsequent functioning, or degradation as we suggest for *S. pneumoniae*. In any case, investigations of species that exhibit different morphology and develop competence at different growth stages will help establish whether location of the transformation machinery at the site of peptidoglycan disruption is a general rule. If so, one would predict physical and possibly functional interaction between components of the transformation and division assemblies.

## Materials and Methods

### Bacterial strains, cultures and transformation

*S. pneumoniae* strains, plasmids and oligonucleotide primers used for PCR and site-directed mutagenesis are listed in Table S1. Stock cultures of pneumococal strains were routinely grown at 37° in Todd–Hewitt (BD Diagnostic System) plus yeast extract (THY) medium to OD_550_ = 0.3; after addition44 of 15% (vol/vol) glycerol, stocks were kept frozen at −70°C. These precultures were used to initiate cultures in C+Y at 6×10^6^ cells mL^−1^. *E. coli* strains used were LE392 and BL21 DE3 pLysS. Transformation was performed as described previously [22].

To monitor transformation proficiency (Figure 1D and Figure 3A), at the indicated times after CSP1 addition 1 mL competent cells were incubated for 3 min at 37°C with 100 ng of R304 chromosomal DNA harboring the *rpsL41* point mutation conferring resistance to streptomycin (Sm^R^). Uptake was terminated by addition of DNase I (50 mg mL^−1^; SIGMA) and incubation was continued for 17 min at 30°C before plating.

To investigate the impact of 5′ terminal label with a single Cy3 dye molecule on transformation (Figure 4A), a 4.2-kb fragment carrying the *rpsL41* Sm^R^ allele was amplified from R304 chromosomal DNA using the OCN79-OCN80 primer pair to generate a Cy3-labelled fragment or the RpsL5-RpsL6 primer pair to generate unlabelled control fragment.

Details of plasmid and strain constructions and transformation procedures are described in Text S2.

### Whole-cell extracts preparation

At the indicated times after CSP1 addition, the OD_550_ was measured (for equivalent loading) and samples (3 mL) were collected by centrifugation. Cell pellets from the CSP-induced and control cultures were stored at −80°C. Whole-cell extracts were prepared by resuspension of cell pellets in 50 µl lysis buffer [10 mM Tris pH 8.0, 1 mM EDTA, 0.01% (wt/vol) DOC, 0.02% (wt/vol) SDS] and incubation at 37°C for 10 min followed by addition of 50 µl loading buffer [0.25 M Tris pH 6.8, 6% (wt/vol) SDS, 10 mM EDTA, 20% (vol/vol) Glycerol] containing 10% (vol/vol) β-mercaptoethanol. Samples were heated for 15 min at 50°C prior to loading.

### Immunoblot analysis

Proteins were separated on pre-cast 4–12% NuPage Bis-Tris gels (Invitrogen) with MOPS-SDS running buffer, transferred to a nitrocellulose membrane using an iBLOT apparatus (Invitrogen) and blocked in 8% (wt/vol) skimmed milk in Tris-buffered saline (TBS) (50 mM Tris-HCl, 150 mM NaCl, pH 8) containing 0.1% (vol/vol) Tween-20. The blocked membrane was probed with anti-GFP, or anti-SsbB antibodies [47]. Primary antibodies were diluted 1∶10,000 into 5% (wt/vol) skimmed milk in TBS supplemented with 0.01% (vol/vol) Tween-20. Primary antibodies were detected using peroxidase-conjugated goat anti-rabbit immunoglobulin G (Sigma) with ECL Western Blotting Detection System (GE Healthcare®) and a luminescent image analyzer (LAS-4000, Fuji). Signals were quantified with a MultiGauge V3.0 Software (Fugifilm).

### Fluorescence microscopy and analysis

Pneumococcal precultures grown in C+Y medium at 37°C to an OD_550_ of 0.06 were induced to develop competence. At indicated times post CSP addition, 1 mL samples were collected, cooled down by addition of 500 µL cold medium, pelleted (3 min, 3,000 g) and resuspended in 50 µL C+Y medium. 2 µL of this suspension were spotted on a microscope slide containing a slab of 1.2% C+Y agarose as described previously [48] before imaging. Images were captured and processed using the Nis-Elements AR software (Nikon) and deconvolution of fluorescent images was carried out using the SVI HuygensEss software v. 4.4 (Scientific Volume Imaging B.V., VB Hilversum, Netherlands).

Images were further analyzed using the MATLAB-based, open-source software MicrobeTracker [21]. For more details, see Text S2.

Cy3-DNA fragments used for microscopy analysis were generated by PCR reaction. The 285-bp, 4.3-kb and 12-kb Cy3-DNA fragments were amplified from R304 chromosomal DNA using the OCN75-OCN76, OCN79-OCN80 and OCN77-OCN78 primer pairs respectively. The 2.2-kb Cy3-DNA fragment encoding the functional *ftsZ*-*gfp* fusion was amplified from strain R3702 with primer pair OMB99-OMB100.

To directly visualize the transformation process, we used strain R3708 harboring the *gfp* orf joined in frame to the 3′ end of *ftsZ* but separated from it by a stop codon (TAA) as a recipient strain. This strain was induced to develop competence and simultaneously incubated for 5 minutes with the 2.2-kb Cy3-DNA fragment containing a functional *ftsZ-gfp* fusion construct (TAA replaced by CTC, coding for Leu). Samples were subsequently spotted on a microscope slide and time lapse experiments were performed capturing images in the red (Cy3) and green (GFP) channels.

## Supporting Information

Figure S1 **Design, construction and evaluation of functionality and impact of GFP-EndA and YFP-EndA fusions.** (**A**) Diagram of the membrane topology of EndA. The *endA* gene encodes a 274 aa protein with a typical but uncleavable signal sequence for membrane transport at its amino end (N-Ter). Based on previous work that indicated that the enzyme retains its signal sequence, which apparently anchors the otherwise extracellular hydrophilic protein to the membrane [23], we fused YFP or GFP to the cytoplasmic N terminus of EndA. IN and OUT refer to the cytoplasm and extracellular space, respectively. The N and C termini of the proteins are shown. H160 corresponds to the conserved first histidine in the H-N-H motif and plays an essential role in catalysis [24], [25]. This residue was changed into an alanine to generate EndA^0^, a protein devoid of nuclease activity (Figure 2D). (**B**) Genomic context of the *yfp*-*endA* fusion integrated at the chromosomal expression platform, CEP [49], and the *gfp*-*endA* construct at the *endA* locus. P, endogenous promoter, P_M_, maltose-inducible promoter. *kan*, Kanamycine resistance gene allowing selection of CEP transformants. (**C**) Functionality of the fusions assessed by measurement of transformation frequency. Using R304 chromosomal DNA as donor, frequencies of Sm^R^ transformants were measured in the wildtype strain R1501 (WT), the *endA* mutant R951, and in strains harboring the GFP-EndA fusion (R2762), the maltose-inducible YFP-EndA fusion (R3243), or the maltose-inducible YFP-EndA fusion together with pMalR, a plasmid encoding the maltose repressor to ensure maximal level of P_M_ repression (R3742). To induce or repress production of YFP-EndA, precultures were prepared in medium supplemented with 1% maltose (*dark grey*) or 0.3% sucrose (*light gray*) respectively as described [49]. Modulation of the level of *yfp-endA* expression (see panel D) did not affect transformation efficiency indicating that N-terminal fusions of fluorescent proteins to EndA are functional and that higher or lower amounts of the protein did not impair transformation proficiency. (**D**) Immunoblot analyses of YFP-EndA and GFP-EndA levels. Cells were grown in C+Y medium supplemented with 1% maltose (M) or 0.3% sucrose (S) to early exponential phase and competence was induced by CSP addition for 10 min. Whole cell extracts were prepared and analyzed by immunoblot using anti-GFP antibodies. The level of the fusion protein is indicated relative to that of GFP-EndA expressed from the endogenous locus. Signals were quantified using a Fujifilm LAS-4000 luminescence image analyzer. Strains used: R3243 (YFP-EndA), R2762 (GFP-EndA) and R1501 (WT). Immunoblots were from the same gel (and the same nitrocellulose membrane) but control lanes were removed for clarity. **Remark**: Full induction with maltose resulted in approximately 7-fold overproduction of the fusion protein whereas repression in the presence of saccharose led to an expression level close to the amount of GFP-EndA expressed from the endogenous locus. We therefore concluded that a 7-fold excess of EndA is not detrimental for transformation. On the other hand, as it was previously established that repression was further increased by 3.2-fold in pMalR containing cells [49], the observation of a transformation frequency in R3742 cells identical to that in wildtype implies that there is at least a 3-fold excess EndA compared to the amount of endonuclease required for full transformation proficiency (i.e., wildtype cell amount). This conclusion is fully consistent with previous biochemical analyses suggesting that only a small fraction (<10%) of EndA molecules is required for DNA uptake [49].
(TIF)

Figure S2 **Subcellular localization and stability of the YFP-EndA fusion protein in strains lacking components of the transformation machinery.** (**A**) Competence-induced clustering of EndA depends on ComX and ComEA. The percent of cells containing YFP-EndA foci 10 minutes after CSP addition is reported for wildtype strain R3243, competent (WT) or noncompetent (WT - CSP), and competent *comX^−^* (R3245), *comGA^−^* (R3247), *comEC^−^* (R3484), *comFA^−^* (R3485) and *comEA^−^* (R3246) mutants. For each strain, data are mean ± s.d. for at least three fields of hundreds cells from a minimum of two independent experiments. (**B**) Distribution of YFP-EndA foci along longitudinal cell axis in *comGA^−^*, *comEC^−^* and *comFA^−^* mutants. See legend of Figure 1C for details. n, number of cells with foci analyzed. (**C**) Immunoblot analyses of YFP-EndA levels in wildtype, *comX^−^*, *comEA^−^*, *comGA^−^*, *comEC^−^* and *comFA^−^* mutants treated with CSP for 10 min. Whole cell extracts were prepared and analyzed by immunoblot using anti-GFP antibodies (*top panel*). As a control for competence induction, the level of the late competence protein SsbB (absent in *comX^−^* mutant) and the constitutively expressed SsbA [47] were monitored using anti-SsbB antibodies (*bottom panel*).
(TIF)

Figure S3 **Evaluation of functionality and stability of protein fusions with fluorescent proteins.** (**A**) Functionality of the fusions assessed by measurement of transformation frequency. See legend of Figure S1C for details. (**B**) Immunoblot analyses of strains containing GFP-ComEA, YFP-EndA, CFP-ComEA and both YFP-EndA and CFP-ComEA fusion proteins. Cells were grown in C+Y medium supplemented with 1% maltose to early exponential phase and competence was induced (+) or not (−) by CSP addition for 10 min. Whole cell extracts were prepared and analyzed by immunoblot using anti-GFP antibodies. Asterisk indicates a degradation product band in cells containing the CFP-ComEA fusion. Positions of MW markers are shown on the right. Immunoblots were from the same gel (and the same nitrocellulose membrane) but control lanes were removed for clarity. Strains used: R1501, WT; R2811, *endA^−^*; R1146, *comEA^−^*; R2940, *gfp*-*comEA*; R3243, *yfp*-*endA*; R3138, *cfp*-*comEA*; R3242, *yfp*-*endA* and *cfp*-*comEA*.
(TIF)

Figure S4 **Post-competence migration of ComEA toward the pole.** (**A**) Correlation between GFP-ComEA foci position (histograms) and transformation proficiency (open diamonds). Re-treatment of the set of data shown in Figure 3A involving recording as polar (green color) foci located in the 0–0.2 interval instead of 0–0.1 (*left*). See legend of Figure 3A for details. Panel on the *right* showing relative frequencies of GFP-ComEA foci at each location reveals a net post-competence trend of ComEA to migrate toward the pole. (**B**) Correlation between YFP-EndA foci position (histograms) and transformation proficiency (open diamonds). Set of data shown in Figure 1D but treated as indicated in panel A for GFP-ComEA. Note that EndA does not exhibit the trend to migrate toward the pole observed for ComEA in panel A.
(TIF)

Figure S5 **Fate of Cy3-DNA fluorescence signal supporting midcell uptake in wildtype cells.** (**A**) Three additional representative cells from the experiment shown in Figure 4B. See legend of Figure 4B for details. (**B**) Time-lapse microscopy of wildtype competent cells following addition of differently sized Cy3-DNA fragments. R1501 cells were treated with CSP for 7 min before addition of the indicated concentrations of DNA and imaging. Due to the speed of DNA internalization, very few DNA dots were observed, some of which appeared associated to cells. Two representative cells are shown for cultures incubated with a 285 bp Cy3-DNA fragment and one cell with a 12 kb Cy3-DNA fragment. Time is indicated in minutes (time 0 is arbitrary). The Cy3 fluorescence images were false colored red and overlaid on phase contrast images. Note that images in panel A were taken with an ImagEM EM-CCD camera, which has better sensitivity but less resolution than the OrcaR2 CCD camera used for panel B. Red arrows point to labile Cy3 signal. Brackets indicate the period during which a focus remains visible. Blue arrows in panel B, middle row (0 and 5 min time points), point to a static fluorescent dot the interpretation of which is problematic. **Remark**: Cy3 fluorescence signals may disappear as a consequence of bleaching or DNA uptake. We favor the latter explanation because the faster disappearance of the 285 bp Cy3-DNA signal compared to the 12 kb Cy3-DNA fragment is fully consistent with the expectations. Based on previous measurement of the rate of DNA uptake [14], the shorter fragment is expected to be internalized within 3 sec and the longer in ∼2 min. Note that two successive DNA dots appear associated to the same cell, at the same location (bottom row in panels A and B), suggesting the possible occurrence of consecutive binding events.
(TIF)

Movie S1 **Dynamic behavior of EndA during competence development.** Fluorescence time-lapse microscopy of competent YFP-EndA cells. Images were acquired every minute for 28 minutes. An arbitrary time point after competence induction within the time-lapse experiment was chosen and set to T = 0. Time is indicated in minutes. Scale bar: 1 µM.
(AVI)

Table S1 **Strains, plasmids and primers used in this study.**
(DOCX)

Text S1 **Supporting results.**
(DOCX)

Text S2 **Supporting materials and methods.**
(DOCX)
