# Supplementary figures and images for: Midcell Recruitment of the DNA Uptake and Virulence Nuclease, EndA, for Pneumococcal Transformation
Source: PLoS Pathog. 2013 Sep 5;9(9):e1003596. doi: 10.1371/journal.ppat.1003596 (PMC3764208; doi:10.1371/journal.ppat.1003596)

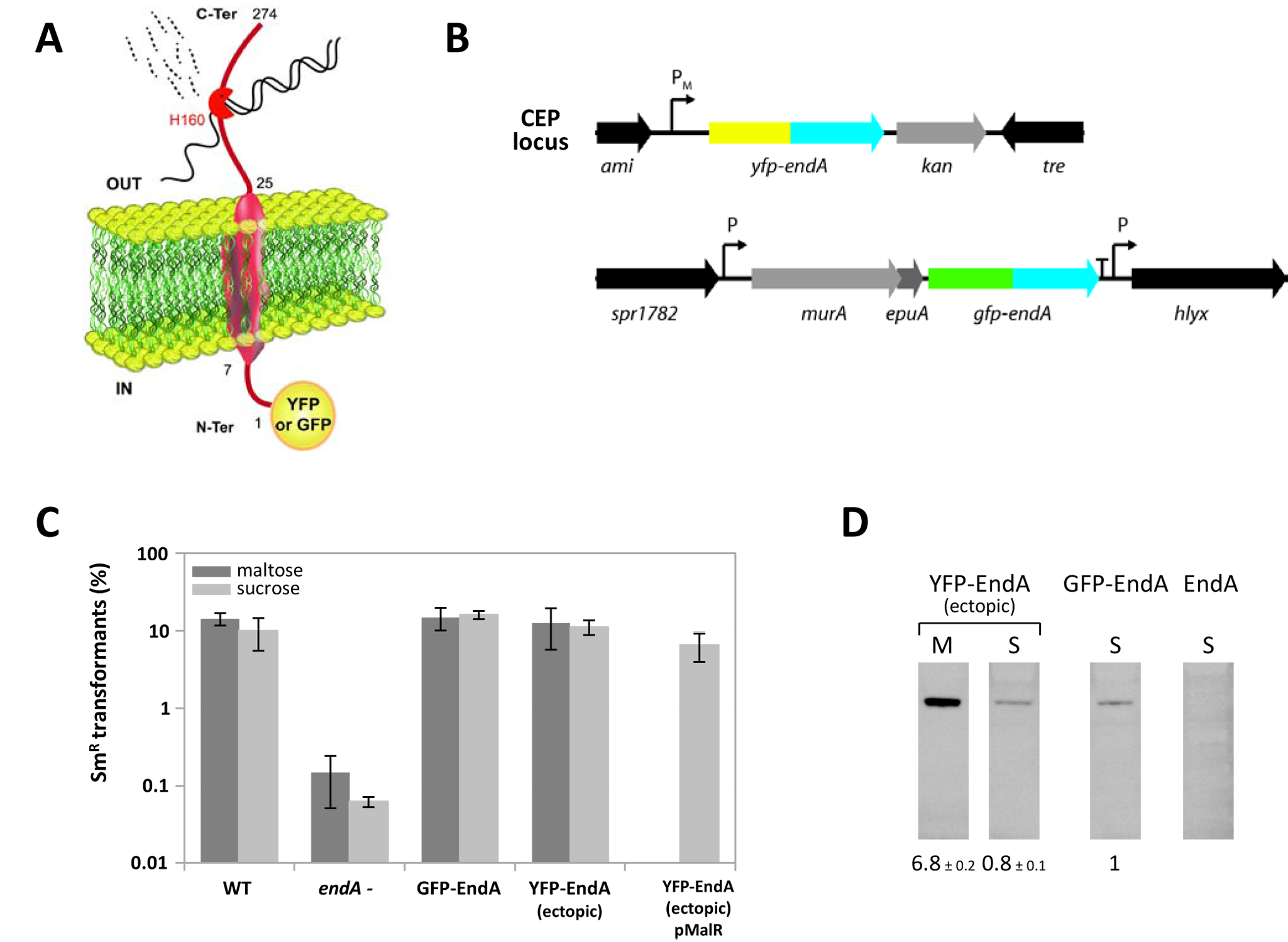

Supplement: Figure S1 — Design, construction and evaluation of functionality and impact of GFP-EndA and YFP-EndA fusions. (A) Diagram of the membrane topology of EndA. The endA gene encodes a 274 aa protein with a typical but uncleavable signal sequence for membrane transport at its amino end (N-Ter). Based on previous work that indicated that the enzyme retains its signal sequence, which apparently anchors the otherwise extracellular hydrophilic protein to the membrane [23], we fused YFP or GFP to the cytoplasmic N terminus of EndA. IN and OUT refer to the cytoplasm and extracellular space, respectively. The N and C termini of the proteins are shown. H160 corresponds to the conserved first histidine in the H-N-H motif and plays an essential role in catalysis [24], [25]. This residue was changed into an alanine to generate EndA0, a protein devoid of nuclease activity (Figure 2D). (B) Genomic context of the yfp-endA fusion integrated at the chromosomal expression platform, CEP [49], and the gfp-endA construct at the endA locus. P, endogenous promoter, PM, maltose-inducible promoter. kan, Kanamycine resistance gene allowing selection of CEP transformants. (C) Functionality of the fusions assessed by measurement of transformation frequency. Using R304 chromosomal DNA as donor, frequencies of SmR transformants were measured in the wildtype strain R1501 (WT), the endA mutant R951, and in strains harboring the GFP-EndA fusion (R2762), the maltose-inducible YFP-EndA fusion (R3243), or the maltose-inducible YFP-EndA fusion together with pMalR, a plasmid encoding the maltose repressor to ensure maximal level of PM repression (R3742). To induce or repress production of YFP-EndA, precultures were prepared in medium supplemented with 1% maltose (dark grey) or 0.3% sucrose (light gray) respectively as described [49]. Modulation of the level of yfp-endA expression (see panel D) did not affect transformation efficiency indicating that N-terminal fusions of fluorescent proteins to EndA are functional and [file ppat.1003596.s001.tif]

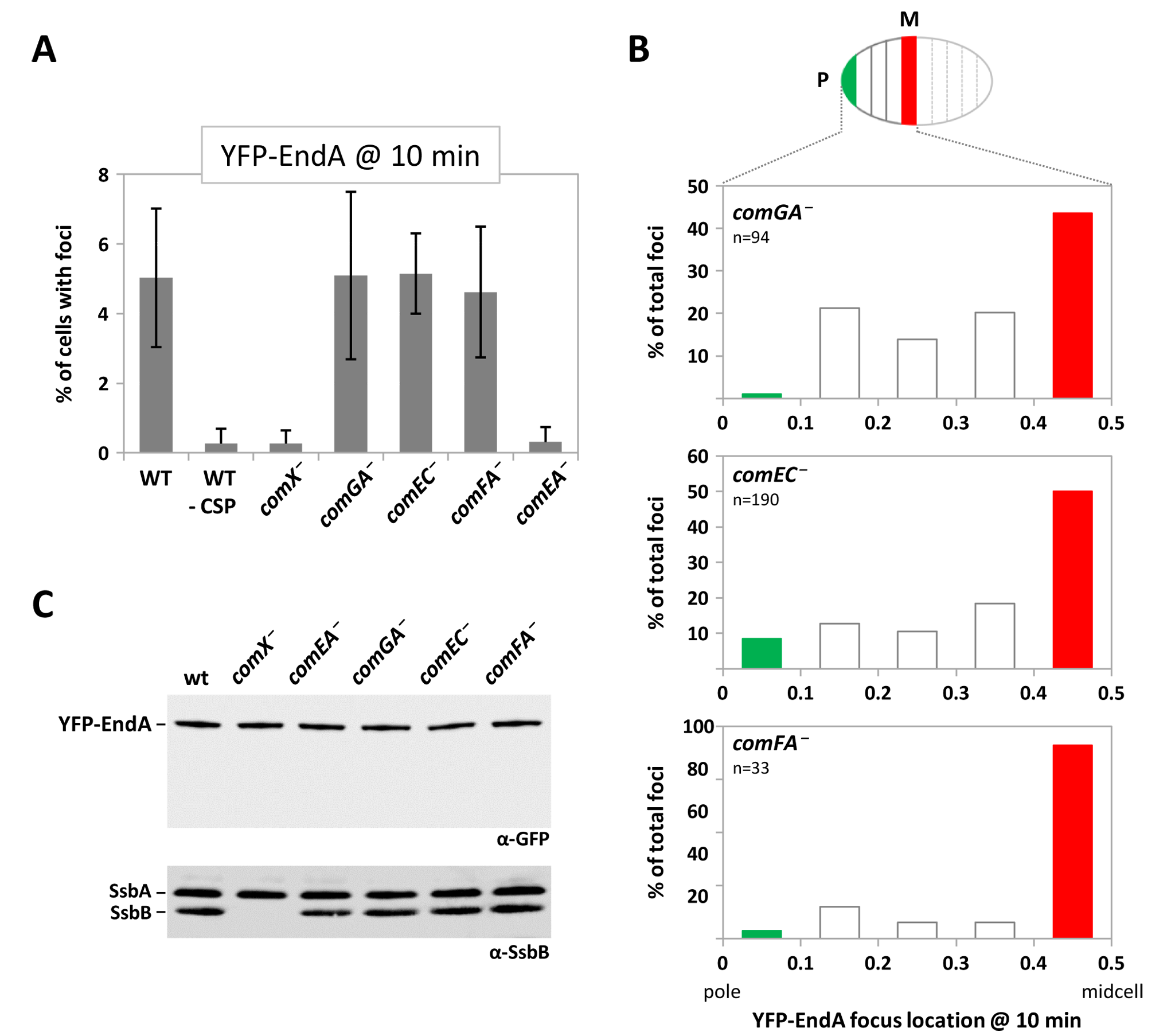

Supplement: Figure S2 — Subcellular localization and stability of the YFP-EndA fusion protein in strains lacking components of the transformation machinery. (A) Competence-induced clustering of EndA depends on ComX and ComEA. The percent of cells containing YFP-EndA foci 10 minutes after CSP addition is reported for wildtype strain R3243, competent (WT) or noncompetent (WT - CSP), and competent comX− (R3245), comGA− (R3247), comEC− (R3484), comFA− (R3485) and comEA− (R3246) mutants. For each strain, data are mean ± s.d. for at least three fields of hundreds cells from a minimum of two independent experiments. (B) Distribution of YFP-EndA foci along longitudinal cell axis in comGA−, comEC− and comFA− mutants. See legend of Figure 1C for details. n, number of cells with foci analyzed. (C) Immunoblot analyses of YFP-EndA levels in wildtype, comX−, comEA−, comGA−, comEC− and comFA− mutants treated with CSP for 10 min. Whole cell extracts were prepared and analyzed by immunoblot using anti-GFP antibodies (top panel). As a control for competence induction, the level of the late competence protein SsbB (absent in comX− mutant) and the constitutively expressed SsbA [47] were monitored using anti-SsbB antibodies (bottom panel). (TIF) [file ppat.1003596.s002.tif]

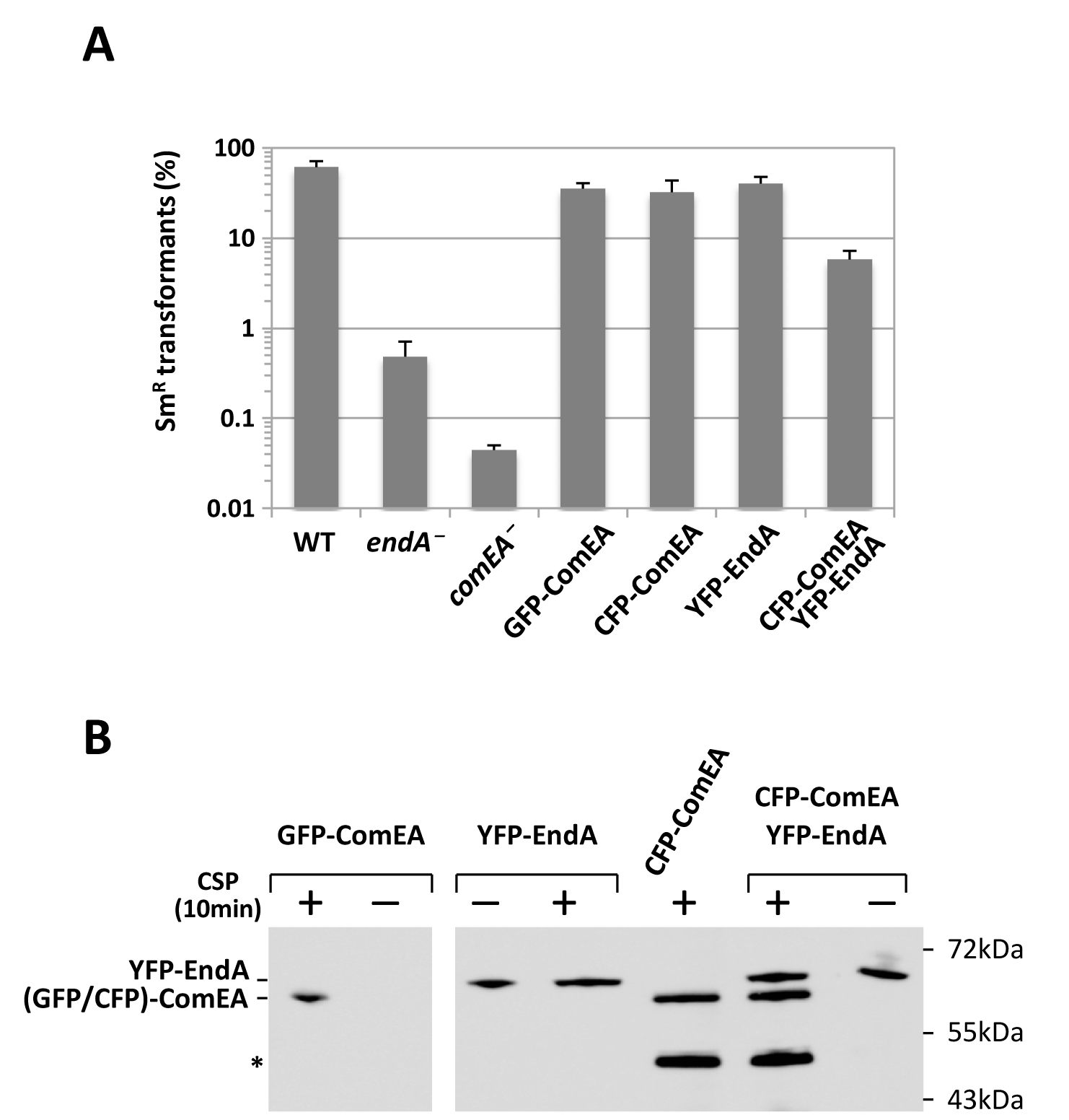

Supplement: Figure S3 — Evaluation of functionality and stability of protein fusions with fluorescent proteins. (A) Functionality of the fusions assessed by measurement of transformation frequency. See legend of Figure S1C for details. (B) Immunoblot analyses of strains containing GFP-ComEA, YFP-EndA, CFP-ComEA and both YFP-EndA and CFP-ComEA fusion proteins. Cells were grown in C+Y medium supplemented with 1% maltose to early exponential phase and competence was induced (+) or not (−) by CSP addition for 10 min. Whole cell extracts were prepared and analyzed by immunoblot using anti-GFP antibodies. Asterisk indicates a degradation product band in cells containing the CFP-ComEA fusion. Positions of MW markers are shown on the right. Immunoblots were from the same gel (and the same nitrocellulose membrane) but control lanes were removed for clarity. Strains used: R1501, WT; R2811, endA−; R1146, comEA−; R2940, gfp-comEA; R3243, yfp-endA; R3138, cfp-comEA; R3242, yfp-endA and cfp-comEA. (TIF) [file ppat.1003596.s003.tif]

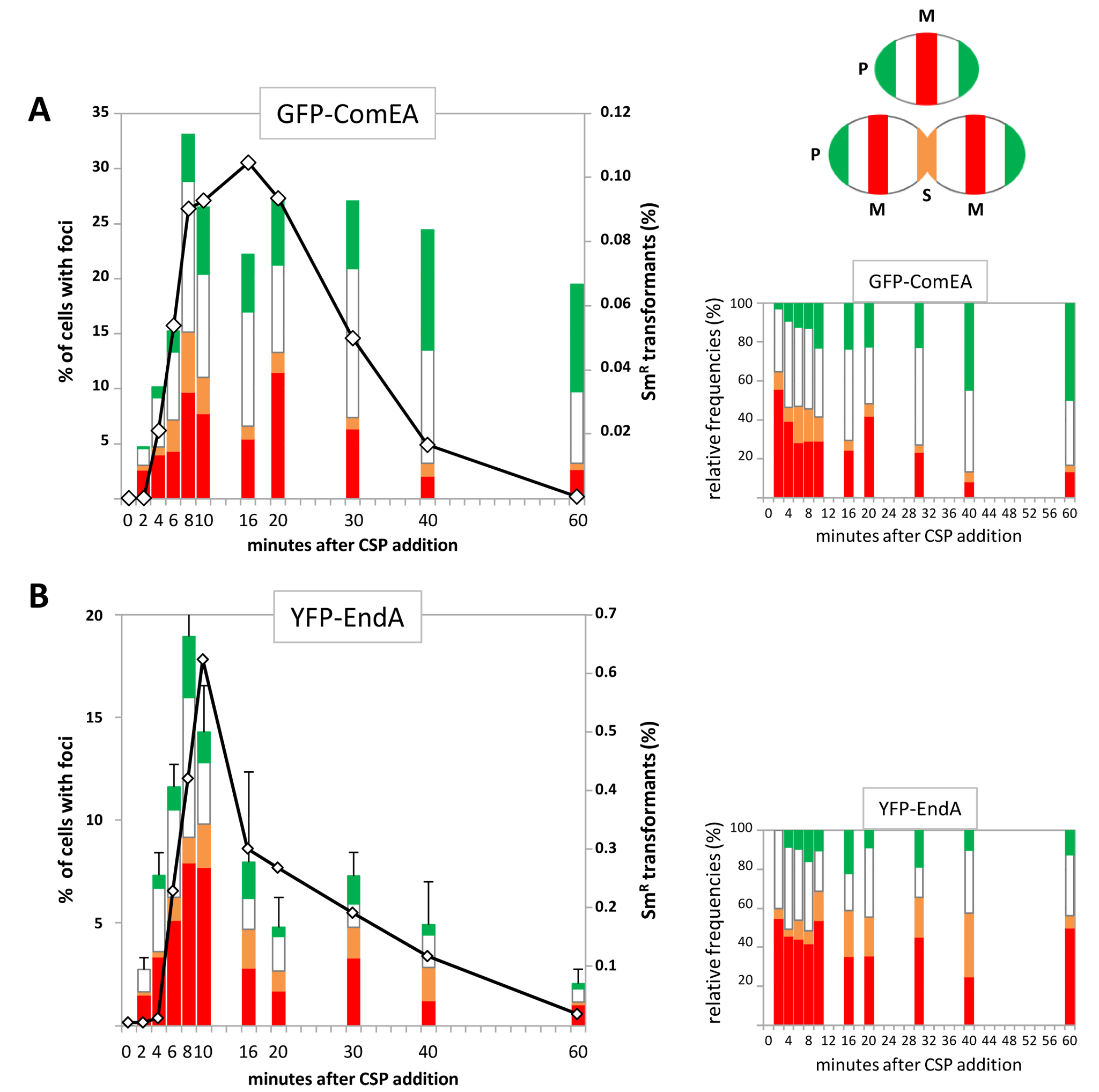

Supplement: Figure S4 — Post-competence migration of ComEA toward the pole. (A) Correlation between GFP-ComEA foci position (histograms) and transformation proficiency (open diamonds). Re-treatment of the set of data shown in Figure 3A involving recording as polar (green color) foci located in the 0–0.2 interval instead of 0–0.1 (left). See legend of Figure 3A for details. Panel on the right showing relative frequencies of GFP-ComEA foci at each location reveals a net post-competence trend of ComEA to migrate toward the pole. (B) Correlation between YFP-EndA foci position (histograms) and transformation proficiency (open diamonds). Set of data shown in Figure 1D but treated as indicated in panel A for GFP-ComEA. Note that EndA does not exhibit the trend to migrate toward the pole observed for ComEA in panel A. (TIF) [file ppat.1003596.s004.tif]

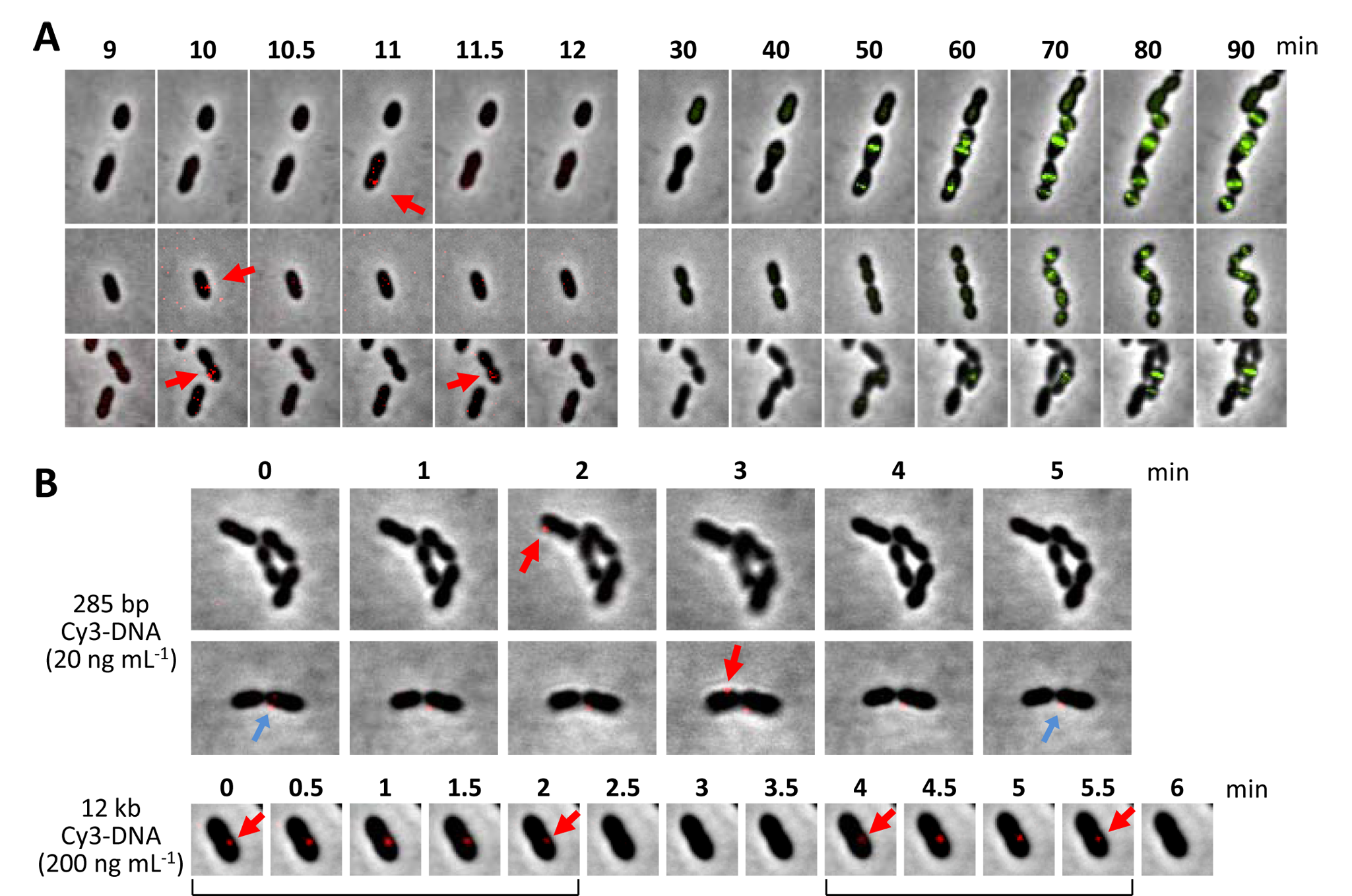

Supplement: Figure S5 — Fate of Cy3-DNA fluorescence signal supporting midcell uptake in wildtype cells. (A) Three additional representative cells from the experiment shown in Figure 4B. See legend of Figure 4B for details. (B) Time-lapse microscopy of wildtype competent cells following addition of differently sized Cy3-DNA fragments. R1501 cells were treated with CSP for 7 min before addition of the indicated concentrations of DNA and imaging. Due to the speed of DNA internalization, very few DNA dots were observed, some of which appeared associated to cells. Two representative cells are shown for cultures incubated with a 285 bp Cy3-DNA fragment and one cell with a 12 kb Cy3-DNA fragment. Time is indicated in minutes (time 0 is arbitrary). The Cy3 fluorescence images were false colored red and overlaid on phase contrast images. Note that images in panel A were taken with an ImagEM EM-CCD camera, which has better sensitivity but less resolution than the OrcaR2 CCD camera used for panel B. Red arrows point to labile Cy3 signal. Brackets indicate the period during which a focus remains visible. Blue arrows in panel B, middle row (0 and 5 min time points), point to a static fluorescent dot the interpretation of which is problematic. Remark: Cy3 fluorescence signals may disappear as a consequence of bleaching or DNA uptake. We favor the latter explanation because the faster disappearance of the 285 bp Cy3-DNA signal compared to the 12 kb Cy3-DNA fragment is fully consistent with the expectations. Based on previous measurement of the rate of DNA uptake [14], the shorter fragment is expected to be internalized within 3 sec and the longer in ∼2 min. Note that two successive DNA dots appear associated to the same cell, at the same location (bottom row in panels A and B), suggesting the possible occurrence of consecutive binding events. (TIF) [file ppat.1003596.s005.tif]
