# Supplementary material for: Midcell Recruitment of the DNA Uptake and Virulence Nuclease, EndA, for Pneumococcal Transformation
Source: PLoS Pathog. 2013 Sep 5;9(9):e1003596. doi: 10.1371/journal.ppat.1003596 (PMC3764208; doi:10.1371/journal.ppat.1003596)
Supplement: Table S1 — Strains, plasmids and primers used in this study. (DOCX) [file ppat.1003596.s007.docx]

| **Strains** | **Genotype**^a^**/description** | | | | **Source/reference** | | | | | | | | |
| --- | --- | --- | --- | --- | --- | --- | --- | --- | --- | --- | --- | --- | --- |
| R800 | | R6 derivative | | | | [[1](#_ENREF_1)] | |  |  |  |  |  |  |
| R304 | | R800 but *nov1*, *rif23*, *str41*; Nov^R^, Rif^R^, Sm^R^ | | | | [[2](#_ENREF_2)] | |  |  |  |  |  |  |
| R825 | | R800 but *comC*::*luc* (*comC*^+^); Ery^R^ | | | | [[3](#_ENREF_3)] | |  |  |  |  |  |  |
| R895 | | R800 but *ssbB*::*luc* (*ssbB*^+^); Cm^R^ | | | | [[4](#_ENREF_4)] | |  |  |  |  |  |  |
| R951 | | R800 but *endA*::*cat8*^C^; Cm^R^ | | | | Claverys strain collection^b^ | |  |  |  |  |  |  |
| R1047 | | R895 but *comA*::*ermAM*^A^; Ery^R^, Cm^R^ | | | | [[3](#_ENREF_3)] | |  |  |  |  |  |  |
| R1062 | | R895 but *comGA*::*spc3*^C^ *mariner* insertion; Ery^R^, Cm^R^, Spc^R^ | | | | [[3](#_ENREF_3)] | |  |  |  |  |  |  |
| R1063 | | R895 but *comFA*::*spc9*^C^ *mariner* insertion; Ery^R^, Cm^R^, Spc^R^ | | | | [[3](#_ENREF_3)] | |  |  |  |  |  |  |
| R1146 | | R895 but *comEA*::*spc6*^A^ *mariner* insertion; Ery^R^, Cm^R^, Spc^R^ | | | | [[3](#_ENREF_3)] | |  |  |  |  |  |  |
| R1501 | | R800 but Δ*comC* | | | | [[5](#_ENREF_5)] | |  |  |  |  |  |  |
| R1818 | | R1501 but *hexA*Δ3::*ermAM*; Ery^R^ | | | | [[6](#_ENREF_6)] | |  |  |  |  |  |  |
| R1960 | | R1501 but *comC*::*luc* (*comC*^+^); Ery^R^ | | | | [[7](#_ENREF_7)] | |  |  |  |  |  |  |
| R2002 | | R1960 but *comX2*::*tet*, *comX1::ermAM*; Cm^R^, Tet^R^, Ery^R^ | | | | [[7](#_ENREF_7)] | |  |  |  |  |  |  |
| R2586 | | R1501 but *comEC*::*ermAM*; Ery^R^ | | | | This study | |  |  |  |  |  |  |
| R2811 | | R1501 but *endA*::*cat8*^C^; Cm^R^ | | | | Claverys strain collection^b^ | |  |  |  |  |  |  |
| R2762 | | R1501 but *gfp-endA* (from plasmid pMB12) | | | | This study | |  |  |  |  |  |  |
| R2940 | | R1501 but *gfp-comEA* (from plasmid pMB14) | | | | This study | |  |  |  |  |  |  |
| R3138 | | R1501 but *cfp-comEA* (from plasmid pMB30) | | | | This study | |  |  |  |  |  |  |
| R3184 | | R1501 but CEP_M_*-yfp-endA* (from plasmid pMB29); Kan^R^ | | | | This study | |  |  |  |  |  |  |
| R3242 | | R3138 but CEP_M_*-yfp-endA* (from R3184); Kan^R^ | | | | This study | |  |  |  |  |  |  |
| R3243 | | R3184 but *endA*::*cat8*^C^; Kan^R^, Cm^R^ | | | | This study | |  |  |  |  |  |  |
| R3244 | | R3142 but *endA*::*cat8*^C^; Kan^R^, Cm^R^ | | | | This study | |  |  |  |  |  |  |
| R3245 | | R3243 but *comX2*::*tet*, *comX1*::*ermAM*; Kan^R^, Cm^R^, Tet^R^, Ery^R^ | | | | This study | |  |  |  |  |  |  |
| R3246 | | R3243 but *comEA*::*spc6*^A^; Kan^R^, Cm^R^, Spc^R^ | | | | This study | |  |  |  |  |  |  |
| R3247 | | R3243 but *comGA*::*spc3*^C^; Kan^R^, Cm^R^, Spc^R^ | | | | This study | |  |  |  |  |  |  |
| R3484 | | R3243 but *comEC*::*ermAM*; Kan^R^, Cm^R^, Ery^R^ | | | | This study | |  |  |  |  |  |  |
| R3485 | | R3243 but *comFA*::*spc9*^C^; Kan^R^, Cm^R^, Spc^R^ | | | | This study | |  |  |  |  |  |  |
| R3606 | | R2940 but *endA*::*cat8*^C^; Cm^R^ | | | | This study | |  |  |  |  |  |  |
| R3702 | | R1818 but *ftsZ-gfp* (from plasmid pMB39); Ery^R^ | | | | This study | |  |  |  |  |  |  |
| R3708 | | R1818 but *ftsZ-*(STOP)*-gfp* (from plasmid pMB40); Ery^R^ | | | | This study | |  |  |  |  |  |  |
| R3740 | | R2811 but *comEA*::*spc6*^A^; Cm^R^, Spc^R^ | | | | This study | |  |  |  |  |  |  |
| R3741 | | R1818 but CEP_M_*-gfp-endA*^H160A^, *endA*::*cat8*^C^; Ery^R^, Kan^R^, Cm^R^ | | | | This study | |  |  |  |  |  |  |
| R3742 | | R3243 but pAPM22 (pMalR); Kan^R^, Cm^R^, Ery^R^ | | | | This study | |  |  |  |  |  |  |
| **Plasmids** | | | |  | | |  | | | |  |  |  |
| pCEP_M_ | | pSC101 derivative carrying the chromosomal platform CEP and the maltose inducible promoter P_M_; Kan^R^ | | | | [[8](#_ENREF_8)] | |  |  |  |  |  |  |
| pUC57-*gfp* (*Sp*) | | pUC57 derivative carrying a 728-bp *Nco*I-*Bam*HI synthetic fragment containing the *gfp*(*Sp*) gene encoding GFP with codon optimized for *S. pneumoniae* R6; Ap^R^ | | | | [[9](#_ENREF_9)] | |  |  |  |  |  |  |
| pUC57-*yfp* (*Sp*) | | pUC57 derivative carrying a 728-bp *Nco*I-*Bam*HI synthetic fragment containing the *yfp*(*Sp*) gene encoding YFP with codon optimized for *S. pneumoniae* R6; Ap^R^ | | | | GenScript, USA | |  |  |  |  |  |  |
| pUC57-*cfp* (*Sp*) | | pUC57 derivative carrying a 728-bp *Nco*I-*Bam*HI synthetic fragment containing the *cfp*(*Sp*) gene encoding CFP with codon optimized for *S. pneumoniae* R6; Ap^R^ | | | | GenScript, USA | |  |  |  |  |  |  |
| pMB12 | | pGBDU derivative carrying the *gfp-endA* fusion; Ap^R^ | | | | This study | |  |  |  |  |  |  |
| pMB14 | | pGBDU derivative carrying the *gfp-comEA* fusion; Ap^R^ | | | | This study | |  |  |  |  |  |  |
| pMB29 | | pCEP_M_ derivative carrying the *yfp-endA* fusion; Kan^R^ | | | | This study | |  |  |  |  |  |  |
| pMB30 | | pGBDU derivative carrying the *cfp-comEA* fusion; Ap^R^ | | | | This study | |  |  |  |  |  |  |
| pMB39 | | pGBDU derivative carrying the *ftsZ-gfp* fusion; Ap^R^ | | | | This study | |  |  |  |  |  |  |
| pMB40 | | pGBDU derivative carrying the *ftsZ-*stop*-gfp* fusion; Ap^R^ | | | | This study | |  |  |  |  |  |  |
| **Primers** | | **Sequence** | | | |  | |  |  |  |  |  |  |
| OMB2 | | GAATTCCCATGGTTTCTAAAGGTG | | | | | |  |  |  |  |  |  |
| OMB4 | | AGACTCGAGGGTTCCGGAATGGTTTCTAAAGGTGAAG | | | | | |  |  |  |  |  |  |
| OMB7 | | CCTGTTCTTAAGAAGGCAGC | | | | | |  |  |  |  |  |  |
| OMB8 | | GGACTTATCAGCCAACCTGTTTAATCCACTGTAACATAGTCTTTAAG | | | | | |  |  |  |  |  |  |
| OMB9 | | ACAGGTTGGCTGATAAGTCC | | | | | |  |  |  |  |  |  |
| OMB10 | | TTATTTCCTCCCGTTAAATAATAG | | | | | |  |  |  |  |  |  |
| OMB11 | | CTATTATTTAACGGGAGGAAATAAGAAGGATAAATGTTGTAGATTAGTG | | | | | |  |  |  |  |  |  |
| OMB12 | | ACAAGACGAGTTTCCAACTCTCTCTCC | | | | | |  |  |  |  |  |  |
| OMB13 | | ccgGGATCCgggagcacaaccgtcccttgattgccaagttacttgaaat | | | | | |  |  |  |  |  |  |
| OMB16 | | ggcAAGCTTcccaggctgtctgaacagcaatgtttttaggattgcttgtt | | | | | |  |  |  |  |  |  |
| OMB17 | | acaattcttcacctttagaaaccatatttctccttatctttagaaaaaggctggt | | | | | |  |  |  |  |  |  |
| OMB18 | | accagcctttttctaaagataaggagaaatatggtttctaaaggtgaagaattgt | | | | | | | |  |  |  |  |
| OMB19 | | ctgtcttgtttttttgttcattccggaacctttatacaattcatccataccatg | | | | | |  |  |  |  |  |  |
| OMB20 | | catggtatggatgaattgtataaaggttccggaatgaacaaaaaaacaagacag | | | | | | | |  |  |  |  |
| OMB26 | | gcgCTGCAGcttgggacgttggggaatagaactaa | | | | | |  |  |  |  |  |  |
| OMB27 | | cctgtaaacaattcttcacctttagaaaccatattttctcctctcttagattattcgt | | | | | | | | | | |  |
| OMB28 | | acgaataatctaagagaggagaaaatatggtttctaaaggtgaagaattgtttacagg | | | | | |  |  |  |  |  |  |
| OMB29 | | tgattttctcgataattgcttccattccggaacctttatacaattcatccatacc | | | | | |  |  |  |  |  |  |
| OMB30 | | ggtatggatgaattgtataaaggttccggaatggaagcaattatcgagaaaatca | | | | | | | | | |  |  |
| OMB31 | | cgcGGATCCcccttgacctgcttgagttcttccaga | | | | | |  |  |  |  |  |  |
| OMB79 | | CGACGGCCGGAACCTTTATACAATTCATCCATACCATGTG | | | | | |  |  |  |  |  |  |
| OMB81 | | tagcctaacaaTGCacctctatcgactgcatgggta | | | | | |  |  |  |  |  |  |
| OMB82 | | tcgatagaggtGCAttgttaggctatgccttaatcggt | | | | | |  |  |  |  |  |  |
| OMB94 | | gcgctgcaggggtgcaggaggtcaacctgaggttggtcgt | | | | | |  |  |  |  |  |  |
| OMB95 | | gtattttcttttacattcatttacttatttatacaattcatccatacc | | | | | |  |  |  |  |  |  |
| OMB96 | | ggtatggatgaattgtataaataagtaaatgaatgtaaaagaaaatac | | | | | |  |  |  |  |  |  |
| OMB97 | | ccagggatcccgaacatctataatgaccttatccgtt | | | | | |  |  |  |  |  |  |
| OMB98 | | cacctccatttttcaaaaatcgttaagagggttccggaatggtttctaa | | | | | |  |  |  |  |  |  |
| OMB99 | | Cy3-gcaggaggtcaacctgaggt | | | | | |  |  |  |  |  |  |
| OMB100 | | | Cy3-Agcctcaaaaggtgccatcgt | | | | | |  |  |  |  |  |
| OCN5 | | tcccggccgtatgaacaaaaaaacaagacagacac | | | | | |  |  |  |  |  |  |
| OCN6 | | gcggatccataagtgacataggagt | | | | | |  |  |  |  |  |  |
| OCN52 | | ctgCtc gag Acg att ttt gaa aaa tgg agg tgt atc ca | | | | | |  |  |  |  |  |  |
| OCN75 | | Cy3-Aattggttcgcaaaccgcgta | | | | | |  |  |  |  |  |  |
| OCN76 | | Cy3-TTACACGTCCACCGCGAAGAA | | | | | |  |  |  |  |  |  |
| OCN77 | | Cy3-Aaattgccgtggcatcaacta | | | | | |  |  |  |  |  |  |
| OCN78 | | Cy3-AAGTGGTGCTGGTTGGGCAT | | | | | |  |  |  |  |  |  |
| OCN79 | | Cy3-Ctggatctacaaaggcggct | | | | | |  |  |  |  |  |  |
| OCN80 | | Cy3-tggctcttcgtcagatgctg | | | | | |  |  |  |  |  |  |
| RpsL5 | | Ctggatctacaaaggcggct | | | |  | |  |  |  |  |  |  |
| RpsL6 | | tggctcttcgtcagatgctg | | | |  | |  |  |  |  |  |  |

^a^ ^R^, resistance; Ap, ampicillin; Ery, erythromycin; Kan, kanamycin; Nov, novobiocin; Rif, rifampicin; Sm, streptomycin; Cm, chloramphenicol; Tet, tetracycline

^b^ *endA*::*cat8*^C^ insertion occurred after positions 97 with respect to the start of the *endA* gene.

**References**

1. Martin B, Prats H, Claverys JP (1985) Cloning of the hexA mismatch-repair gene of Streptococcus pneumoniae and identification of the product. Gene 34: 293-303.

2. Mortier-Barriere I, de Saizieu A, Claverys JP, Martin B (1998) Competence-specific induction of recA is required for full recombination proficiency during transformation in Streptococcus pneumoniae. Mol Microbiol 27: 159-170.

3. Berge M, Moscoso M, Prudhomme M, Martin B, Claverys JP (2002) Uptake of transforming DNA in Gram-positive bacteria: a view from Streptococcus pneumoniae. Mol Microbiol 45: 411-421.

4. Chastanet A, Prudhomme M, Claverys JP, Msadek T (2001) Regulation of Streptococcus pneumoniae clp genes and their role in competence development and stress survival. J Bacteriol 183: 7295-7307.

5. Dagkessamanskaia A, Moscoso M, Henard V, Guiral S, Overweg K, et al. (2004) Interconnection of competence, stress and CiaR regulons in Streptococcus pneumoniae: competence triggers stationary phase autolysis of ciaR mutant cells. Mol Microbiol 51: 1071-1086.

6. Caymaris S, Bootsma HJ, Martin B, Hermans PW, Prudhomme M, et al. (2010) The global nutritional regulator CodY is an essential protein in the human pathogen Streptococcus pneumoniae. Mol Microbiol 78: 344-360.

7. Martin B, Soulet AL, Mirouze N, Prudhomme M, Mortier-Barriere I, et al. (2013) ComE/ComE~P interplay dictates activation or extinction status of pneumococcal X-state (competence). Mol Microbiol 87: 394-411.

8. Guiral S, Henard V, Laaberki MH, Granadel C, Prudhomme M, et al. (2006) Construction and evaluation of a chromosomal expression platform (CEP) for ectopic, maltose-driven gene expression in Streptococcus pneumoniae. Microbiology 152: 343-349.

9. Martin B, Granadel C, Campo N, Henard V, Prudhomme M, et al. (2010) Expression and maintenance of ComD-ComE, the two-component signal-transduction system that controls competence of Streptococcus pneumoniae. Mol Microbiol 75: 1513-1528.
