# Supplementary material for: Midcell Recruitment of the DNA Uptake and Virulence Nuclease, EndA, for Pneumococcal Transformation
Source: PLoS Pathog. 2013 Sep 5;9(9):e1003596. doi: 10.1371/journal.ppat.1003596 (PMC3764208; doi:10.1371/journal.ppat.1003596)
Supplement: Text S1 — Supporting results. (DOCX) [file ppat.1003596.s008.docx]

**Functional Interferences Between ComEA and EndA Fusion Proteins.** The possibility of a direct interaction of EndA and ComEA was indirectly supported by the finding that a functional YFP-EndA fusion which clustered normally at competence in wildtype cells failed to do so in cells harboring a CFP-ComEA fusion. In addition, double-fusion cells exhibited a ~10-fold reduction in transformation frequency, while single-fusion cells exhibited wildtype transformation frequencies despite the presence of similar amounts of each fusion protein (Figure S3, A and B). We tentatively attribute these effects to impairment of ComEA-EndA functional interaction in cells harboring both YFP-EndA and CFP-ComEA fusions.
