# Supplementary material for: Midcell Recruitment of the DNA Uptake and Virulence Nuclease, EndA, for Pneumococcal Transformation
Source: PLoS Pathog. 2013 Sep 5;9(9):e1003596. doi: 10.1371/journal.ppat.1003596 (PMC3764208; doi:10.1371/journal.ppat.1003596)
Supplement: Text S2 — Supporting materials and methods. (DOCX) [file ppat.1003596.s009.docx]

**Pneumococcal Transformation.** CSP-induced transformation was performed in C+Y medium as described previously [[1](#_ENREF_1)], using precompetent cells treated at 37°C for 10 minutes with synthetic CSP1 (25 ng mL^−1^). After addition of transforming DNA and unless otherwise indicated, cells were incubated for 20 min at 30°C. Transformants were selected by plating on CAT-agar supplemented with 4% (vol/vol) horse blood, followed by selection using a 10 mL overlay containing chloramphenicol (Cm; 4.5 µg mL^−1^), erythromycin (Ery; 0.05 µg mL^−1^), kanamycin (Kan; 250 µg mL^−1^), spectinomycin (Spc; 100 µg mL^−1^), Sm (200 µg mL^−1^) or tetracyclin (Tc; 1 µg mL^−1^), after phenotypic expression for 120 at 37°C.

**Plasmid and Strain Constructions.** Strain R2762 harboring the *gfp*-*endA* fusion at the *endA* endogenous locus was obtained by transformation of strain R1501 with a ligation mixture containing the pMB12 (i.e., pGBDU-*gfp*-*endA*) construct. To generate pMB12, primers were designed to amplify a region upstream of *endA* (primer pair OMB13 and OMB17, and R304 DNA as template), a downstream region containing *endA* orf (primer pair OMB20 and OMB16, and R304 DNA as template) and the *gfp* gene (primer pair OMB18 and OMB19, and template DNA pUC57-*gfp*(*Sp*) [[1](#_ENREF_1)]. The PCR products were gel-purified and used as templates in a SOEing PCR using the outer primers OMB13 and OMB16, yielding a PCR product containing the *gfp*-*endA* fusion. The PCR product was subsequently cut with *Bam*HI and *Hind*III, and ligated with the pGBDU plasmid also cut with *Bam*HI and *Hind*III.

Strains R2940 and R3138 containing the *gfp*-*comEA* and *cfp*-*comEA* fusions at the *comEA* endogenous locus were obtained by transformation of strain R1501 with constructs pMB14 and pMB30. To generate the pMB14 construct (i.e., pGBDU-*gfp*-*comEA*), primers were designed to amplify an upstream region containing the promoter of *comEA-comEC* operon (primer pair OMB26 and OMB27, and R304 DNA as template), a downstream region containing the *comEA* open reading frame (primer pair OMB30 and OMB31, and R304 DNA from as template) and the *gfp* gene (primer pair OMB28 and OMB29, and template DNA pUC57-*gfp*(*Sp*) [[1](#_ENREF_1)]. The PCR products were gel-purified and used as templates in a SOEing PCR using the outer primers OMB26 and OMB31, yielding a PCR product containing the *gfp*-*comEA* fusion under the control of the *comEA* promoter. The PCR product was subsequently cut with *Pst*I and *Bam*HI, and ligated with the pGBDU plasmid also cut with *Pst*I and *Bam*HI. The ligation mixture was directly used for transformation of pneumococcal cells. The pMB30 construct (i.e., pGBDU-*cfp*-*comEA*) was obtained as pMB14 except that a PCR product containing the *cfp* gene (instead of *gfp*) was generated using oligonucleotides OMB28 and OMB29, and template DNA pUC57-*cfp*(*Sp*). Plasmid pUC57-*cfp*(*Sp*) contains the gene encoding eCFP (Clonetech) resynthesized with codons optimized for *S. pneumoniae* strain R6 (http://gib.genes.nig.ac.jp/) using the OptimumGene™ algorithm and cloned into pUC57 by Genscript USA. Notably, in addition to codon optimization, two modifications were included during synthesis: a nucleotide transition at position 171 (A->T), to inactivate an internal *Nco*I restriction site, and an amino acid substitution (A206K) that prevents CFP dimerization [[2](#_ENREF_2)].

To achieve ectopic expression of the *yfp-endA* fusion in *S. pneumoniae*, we constructed a derivative of pCEP_M_, an integrative plasmid that allows chromosomal integration of a gene at CEP and its expression under the control of the maltose inducible promoter P_M_ [[3](#_ENREF_3)]. Plasmid pMB29 (i.e., pCEP_M_-*yfp*-*endA*) was created in a three-way ligation with an *Eag*I*-Bam*HI PCR product containing *endA* orf (oligonucleotide OCN5 and OCN6, and R304 DNA as template), a *Nco*I-*Eag*I PCR product containing the *yfp* gene with codons optimized for *S. pneumoniae* (oligonucleotide OMB2 and OMB79, and template DNA pUC57-*yfp*(*Sp*)), and pCEP_M_ cut with *Nco*I and *Bam*HI. The pUC57-*yfp*(*Sp*) plasmid contains a *yfp* variant derived from pUC57-*gfp*(*Sp*) [[1](#_ENREF_1)] with a single point mutation (T203Y) generated by Genscript USA.

Construction of strain R3741 encoding the GFP-EndA^H160A^ fusion at CEP under P_M_ was first achieved by replacing the *yfp* gene from the *yfp*-*endA* fusion by the *gfp* variant by transformation of strain R3243 with plasmid pUC57-*gfp(Sp)*. Presence of mutation Y203T in *gfp* was confirmed by sequencing the *gfp-endA* orf. Then, to introduce the EndA^H160A^ mutation, primers were designed to amplify the 5’ and the 3’ regions of *endA*, both including the H160A mutation (primer pairs OCN5 and OMB81 for the 5’ region and OMB86 and OCN6 for the 3’ region, and R304 DNA as template). The PCR products were gel-purified and used as templates in a SOEing PCR using the outer primers OCN5 and OCN6, yielding a PCR product containing the *endA*^H160A^ mutant gene*.* This product was directly used to transform pneumococcal cells. Presence of mutation H160A in *endA* (and Y203T in *gfp*) were confirmed by sequencing the entire *gfp-endAH160A* coding region.

Strain R3708 harboring the *gfp* orf fused in frame to the 3’ end of *ftsZ*, but with a stop codon inserted between the two orfs was engineered as follows. First a strain (R3702) harboring a functional *ftsZ-gfp* fusion was constructed by transformation of strain R1501 with plasmid pMB39 resulting in integration of the *ftsZ*-*gfp* construct at the *ftsZ* endogenous locus. To generate plasmid pMB39, primers were designed to amplify *ftsZ* (primer pair OMB94 and OCN52, and R304 DNA as template), the *gfp* orf together with a linker at its 5’ extremity (primer pair OMB4 and OMB95, and template DNA pUC57-*gfp*(*Sp*)) and the *ftsZ* downstream chromosomal region (primer pair OMB96 and OMB97, and R304 DNA as template). The PCR products containing the *ftsZ* and *gfp* genes were subsequently cut with *Xho*I and ligated. The ligation and the OMB96-OMB97 PCR products were gel-purified and used as templates in a SOEing PCR using the outer primers OMB94 and OMB97, yielding a PCR product comprising the *ftsZ-gfp* fusion and the downstream chromosomal region of *ftsZ*. The resulting PCR product was subsequently cut with *Pst*I and *Bam*HI, and ligated with the pGBDU plasmid also cut with *Pst*I and *Bam*HI. Insertion of a stop codon between the *ftsZ* and *gfp* genes was then achieved by site-directed mutagenesis [[4](#_ENREF_4)] using primer OMB98 and pMB39 as template generating plasmid pMB40. Plasmids pMB39 and pMB40 were used to transform the *S. pneumoniae* strains R1818 without selection as previously described [[5](#_ENREF_5)] to generate strain R3702 and R3708 respectively. Note that the resulting strains contain a *hexA* mutation negating any effect of the mismatch repair system on transformation efficiencies [[6](#_ENREF_6)].

Construction of strain R2586 harboring a deletion of the *comEC* orf was constructed by amplifying an upstream region containing the promoter of the *comEA-comEC* operon (primer pair OMB7 and OMB8, and R304 DNA as template), the downstream chromosomal region of *comEC* (primer pair OMB11 and OMB12, and R304 DNA from as template) and the *ermAM* gene encoding resistance to Ery (primer pair OMB9 and OMB10, and template pR408 [[7](#_ENREF_7)]). The PCR products were gel-purified and used as templates in a SOEing PCR using the outer primers OMB7 and OMB12, yielding a PCR product containing the *comEA-comEC* operon with *ermAM* in place of *comEC*. The PCR product was directly used for transformation of pneumococcal cells R1501, selecting for Ery^R^ transformants.

**Fluorescence Microscopy and Analysis.** After gentle thawing of stock cultures, aliquots were inoculated at OD_550_ = 0.006 in C+Y medium and grown at 37°C to an OD_550_ of 0.3. These precultures were inoculated (1/100) in C+Y medium and incubated at 37°C to an OD_550_ of 0.06. Then, competence was induced with synthetic CSP1 (25 ng mL^−1^). At different times after CSP addition, 1 mL samples were collected, cooled down by addition of 500 µL cold C+Y medium, pelleted (3 min, 3,000 g) and resuspended in 50 µL C+Y medium. Two μL of this suspension were spotted on a microscope slide containing a slab of 1.2% C+Y agarose as described previously [[8](#_ENREF_8)].

Phase contrast and fluorescence microscopy were performed with an automated inverted epifluorescence microscope Nikon Ti-E/B equipped with the “perfect focus system” (PFS, Nikon), a phase contrast objective (CFI Plan Fluor DLL 100X oil NA1.3), Semrock filters sets for GFP (Ex: 482BP35; DM: 506; Em: 536BP40), YFP (Ex: 500BP24; DM: 520; Em: 542BP27), Cy3 (Ex: 531BP40; DM: 562; Em: 593BP40), a Nikon Intensilight 130W High-Pressure Mercury Lamp, and a monochrome OrcaR2 digital CCD camera (Hamamatsu) or an ImageEM EMCCD camera (Hamamatsu). The microscope is equipped with a chamber thermostated at 30°C. For time-lapse experiments, temperature was set at 37°C. All fluorescence images were acquired with a minimal exposure time to minimize bleaching and phototoxicity effects. Fluorescence images were captured and processed using Nis-Elements AR software (Nikon). YFP, GFP and Cy3 fluorescence images were respectively false colored yellow, green and red, and overlaid on phase contrast images. Two-dimensionnal deconvolution was carried out on fluorescent images using the SVI HuygensEss software v. 4.4 (Scientific Volume Imaging B.V., VB Hilversum, Netherlands). Here we used an algorithm based on the Classic Maximum Likelihood Estimator running for 15 iterations and a signal to noise ratio of 30.

Images were analyzed with the MATLAB-based open-source software MicrobeTracker [[9](#_ENREF_9)]. Cell contours were obtained using the *alg4 ecoli2* algorithm implemented in MicrobeTracker and parameters spliltTreshold, joindist and joinangle were refined to fit the shape of *S. pneumoniae*. Fluorescent foci inside the cells were automatically identified using the SpotFinder tool from MicrobeTracker. For each experiment, four fields of several hundred cells, each from three independent experiments, were analyzed. Occasionally, SpotFinder could also detect cells with foci in noncompetent cultures (3 cells over a total of 2,823, Fig. 1*B*). A closer examination of these cells revealed that the shape of the foci were irregular and generally less bright than the ones found in competent cells suggesting that these signals were false positive due to a higher concentration of YFP-EndA fluorescence at the septum which is composed of a double membrane. To plot the distribution of fluorescent foci along longitudinal cell axis, distances of foci from one pole were recorded as a function of cell length in relative coordinates extending from 0 at the pole to 0.5 at midcell. The distributions were plotted as the percentage of total foci versus cell length. All statistics were calculated using Graphpad Prism 4. Statistical significance was determined using a two-tailed Mann-Whitney test (p = 0.0003).

**Supporting References**

1. Martin B, Granadel C, Campo N, Henard V, Prudhomme M, et al. (2010) Expression and maintenance of ComD-ComE, the two-component signal-transduction system that controls competence of Streptococcus pneumoniae. Mol Microbiol 75: 1513-1528.

2. Zacharias DA, Violin JD, Newton AC, Tsien RY (2002) Partitioning of lipid-modified monomeric GFPs into membrane microdomains of live cells. Science 296: 913-916.

3. Guiral S, Henard V, Laaberki MH, Granadel C, Prudhomme M, et al. (2006) Construction and evaluation of a chromosomal expression platform (CEP) for ectopic, maltose-driven gene expression in Streptococcus pneumoniae. Microbiology 152: 343-349.

4. Shenoy AR, Visweswariah SS (2003) Site-directed mutagenesis using a single mutagenic oligonucleotide and DpnI digestion of template DNA. Anal Biochem 319: 335-336.

5. Quevillon-Cheruel S, Campo N, Mirouze N, Mortier-Barriere I, Brooks MA, et al. (2012) Structure-function analysis of pneumococcal DprA protein reveals that dimerization is crucial for loading RecA recombinase onto DNA during transformation. Proc Natl Acad Sci U S A.

6. Claverys JP, Lacks SA (1986) Heteroduplex deoxyribonucleic acid base mismatch repair in bacteria. Microbiol Rev 50: 133-165.

7. Prudhomme M (2007) In vitro mariner mutagenesis of Streptococcus pneumoniae: tools and traps. In: Hakenbeck R, editor. The Molecular Biology of Streptococci. Norwich, UK: Horizon Scientific Press. pp. 511-517.

8. de Jong IG, Beilharz K, Kuipers OP, Veening JW (2011) Live Cell Imaging of Bacillus subtilis and Streptococcus pneumoniae using Automated Time-lapse Microscopy. J Vis Exp.

9. Sliusarenko O, Heinritz J, Emonet T, Jacobs-Wagner C (2011) High-throughput, subpixel precision analysis of bacterial morphogenesis and intracellular spatio-temporal dynamics. Mol Microbiol 80: 612-627.
